# Modeling Neurodevelopmental Disorders and Epilepsy Caused by Loss of Function of *kif2a* in Zebrafish

**DOI:** 10.1523/ENEURO.0055-21.2021

**Published:** 2021-09-07

**Authors:** Michèle Partoens, Ann-Sofie De Meulemeester, Hoi-Khoanh Giong, Duc-Hung Pham, Jeong-Soo Lee, Peter A. de Witte, Aleksandra Siekierska

**Affiliations:** 1 Laboratory for Molecular Biodiscovery, Department of Pharmaceutical and Pharmacological Sciences, KU Leuven, 3000 Leuven, Belgium; 2 Disease Target Structure Research Centre, Korea Research Institute of Bioscience and Biotechnology, Daejeon 34141, Republic of Korea; 3 KRIBB School, University of Science and Technology, Daejeon 34141, Republic of Korea; 4 Dementia DTC R&D Convergence Program, Korea Institute of Science and Technology, Seoul 02792, Republic of Korea

**Keywords:** drug-resistant epilepsy, KIF2A, malformations of cortical development, seizures, zebrafish

## Abstract

In recent years there has been extensive research on malformations of cortical development (MCDs) that result in clinical features like developmental delay, intellectual disability, and drug-resistant epilepsy (DRE). Various studies highlighted the contribution of microtubule-associated genes (including tubulin and kinesin encoding genes) in MCD development. It has been reported that *de novo* mutations in *KIF2A*, a member of the *kinesin-13* family, are linked to brain malformations and DRE. Although it is known that KIF2A functions by regulating microtubule depolymerization via an ATP-driven process, *in vivo* implications of *KIF2A* loss of function remain partly unclear. Here, we present a novel *kif2a* knock-out zebrafish model, showing hypoactivity, habituation deficits, pentylenetetrazole-induced seizure susceptibility and microcephaly, as well as neuronal cell proliferation defects and increased apoptosis. Interestingly, *kif2a*^−/−^ larvae survived until adulthood and were fertile. Notably, our *kif2a* zebrafish knock-out model demonstrated many phenotypic similarities to *KIF2A* mouse models. This study provides valuable insights into the functional importance of *kif2a* in zebrafish and phenotypical hallmarks related to *KIF2A* mutations. Ultimately, this model could be used in a future search for more effective therapies that alleviate the clinical symptoms typically associated with MCDs.

## Visual Abstract

**Figure:**
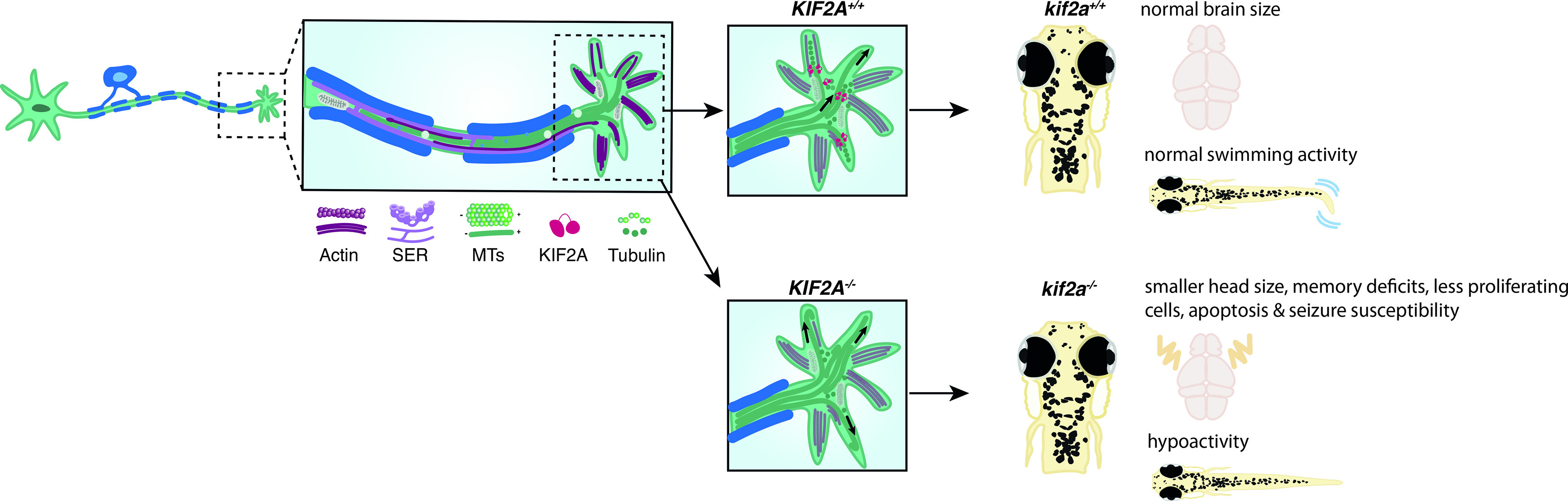

## Significance Statement

This study addresses the functional importance of Kif2a and the phenotypical hallmarks related to *KIF2A* mutations in zebrafish. We generated a novel *kif2a* loss-of-function model in zebrafish and demonstrated that Kif2a deficiency was associated with behavioral alterations, habituation deficits, enhanced susceptibility to seizures, microcephaly, neuronal cell proliferation defects, and increased apoptosis. These findings provide insights into a better understanding of the *kif2a*-related pathologic mechanisms and, eventually, might help in the search for novel and more effective medications to alleviate clinical symptoms typically associated with MCDs.

## Introduction

In humans, proper cortical development is highly dependent on the following three primary time-dependent phases: proliferation of the pool of progenitor cells, along with neuronal migration in the cortical plate, and, ultimately, their differentiation (Diaz and Gleeson, 2009; Sun et al., 2017; Buchsbaum and Cappello, 2019). Alterations in any of these series of orchestrated events may result in a disrupted neuronal circuitry and predispose an individual to the development of neurodevelopmental disorders including malformations of cortical development (MCDs; Leventer et al., 2008; Buchsbaum and Cappello, 2019). MCDs are increasingly recognized as a cause of developmental delay, intellectual disability and drug-resistant epilepsy (DRE), and can be caused by various underlying genetic or exogenous factors (Pang et al., 2008; Buchsbaum and Cappello, 2019). Recent advances in genetics have led to the discovery of a number of new genes associated with MCDs. Among these are kinesin superfamily genes (*KIFs*), encoding ATP-driven microtubule-associated proteins that use the energy of ATP hydrolysis to carry out mechanical work along microtubule tracks (Poirier et al., 2013; Ali and Yang, 2020). It has been reported that *de novo* mutations in kinesin family member 2a (*KIF2A*) represent a significant cause of lissencephaly, microcephaly, and DRE (Bahi-Buisson and Cavallin, 2016; Homma et al., 2003; Poirier et al., 2013; Guerrini and Dobyns, 2014; Tian et al., 2016; Cavallin et al., 2017; Broix et al., 2018). *KIF2A* belongs to the human *kinesin-13* family (M-kinesins) consisting of *KIF2A*, *KIF2B*, *KIF2C/MCAK*, and *KIF24* (Miki et al., 2005), and is highly abundant in developing neurons being involved in neuronal migration, axonal elongation, and pruning (Homma et al., 2003, 2018; Maor-Nof et al., 2013; Broix et al., 2018). Several studies demonstrated that multiple *KIF2A* mutations found in patients are located in or near the ATP binding site of the kinesin motor domain (residues p.S317, p.T320, and p.H321; Poirier et al., 2013; Guerrini and Dobyns, 2014; Tian et al., 2016; Cavallin et al., 2017), thus affecting ATP hydrolysis or microtubule binding, ultimately resulting in a nonfunctional kinesin (Poirier et al., 2013).

Since epilepsy is the most common clinical consequence of MCDs (Leventer et al., 1999, 2008; Guerrini and Dobyns, 2014; Tian et al., 2016; Cavallin et al., 2017), antiepileptic drugs are used to control seizures; however, many patients remain unresponsive to the medication therapy (Represa, 2019). Hence, there is an unmet medical need to advance our understanding of the pathogenesis of MCDs and related epilepsies. In recent years, mouse *KIF2A* models have been developed, which has led to important insights concerning cortical defects and epilepsy (Homma et al., 2003, 2018; Gilet et al., 2020). *Kif2a*-null mice showed multiple neurodevelopmental abnormalities like early postnatal death, cortical lamination defects as a result of aberrant neuronal migration, and lateral cortical ventricle enlargement; however, no manifestation of epileptic seizures could be reported (Homma et al., 2003). Further characterization of the disease phenotype was allowed by a tamoxifen-inducible *Kif2a* conditional knock-out (KO) mouse model, demonstrating the role of KIF2A in the precise postnatal hippocampal wiring (Homma et al., 2018). Although neuronal proliferation or migration was not disrupted, mice displayed severe epilepsy, likely a cause of death at 6 weeks postbirth (Homma et al., 2018). Recently, a conditional knock-in (KI) mouse model was developed, bearing the human *KIF2A* p.H321D missense point variant identified in MCD patients (Gilet et al., 2020). *KIF2A^+/H321D^* KI mice survived and displayed microcephaly and neuroanatomical anomalies like hippocampal structure abnormalities and cortical layer disorganization, resulting from abnormal neuronal migration and increased cell death (Gilet et al., 2020). Moreover, *KIF2A*-deficient animals showed behavioral deficits and susceptibility to epilepsy, correlating with the described human phenotype.

Over the years, the zebrafish has emerged as a promising model to study neurodevelopmental diseases and their pathologic processes in early development. The developing larval zebrafish brain presents numerous similar structures and cell types that are found in mammals (Kalueff et al., 2014). Moreover, their small size, high fecundity, *ex utero* development, and genetic amenability allow for higher-throughput screenings, improving CNS drug discovery (Stewart et al., 2015; Khan et al., 2017).

This study intends to advance our understanding of *KIF2A*-related MCDs by investigating the pathologic consequences of Kif2a loss of function *in vivo* in a novel zebrafish model. By means of neurobehavioral and electrophysiological assays, we demonstrated that a loss of Kif2a in zebrafish causes behavioral alterations like hypoactivity and habituation deficits, reduced head size, neuronal cell proliferation defects, apoptosis, and, importantly, increased pentylenetetrazole (PTZ)-induced seizure susceptibility. Despite these abnormalities, *kif2a*^−/−^ larvae survived until adulthood and were fertile. Altogether, our results show that the *kif2a* KO model could ultimately be used in the search for novel and more effective medications that alleviate the clinical symptoms associated with MCDs.

## Materials and Methods

### Zebrafish husbandry

All zebrafish (*Danio rerio*) strains used in this study were maintained at 28 ± 2°C on a 14:10 h light/dark cycle under standard aquaculture conditions in a UV-sterilized rack recirculating system equipped with a mechanical and biological filtration unit. Embryos were collected via natural spawning and immediately transferred to Danieau’s medium (0.3× Danieau’s medium), containing 1.5 mm HEPES buffer pH 7.2, 17.4 mm NaCl, 0.21 mm KCl, 0.18 mm Ca(NO_3_)_2_, 0.12 mm MgSO_4_, and 0.6 μm methylene blue. For the experiments, embryos and larvae were kept at 28.5°C with a photoperiod of 14 h. In all experiments, zebrafish larvae were treated in a humane way and with regard for alleviation of suffering. All zebrafish experiments were performed in accordance with the guidelines of and approval by the Ethical Committee of the University of Leuven (P023/2017 and P027/2019) and by the Belgian Federal Department of Public Health, Food Safety and Environment (LA1210199).

### Generation of the *kif2a* CRISPR knock-out zebrafish line

A *kif2a* knock-out line was generated via the CRISPR/Cas9 technique (Hruscha et al., 2013; Hwang et al., 2013). *kif2a* single-guide RNA (sgRNA) targeting exon 5 in the *kif2a* gene (5′-CAGCCAGAATCAGCACCCCC-3′) was designed using the CHOP CHOP web tool (https://chopchop.cbu.uib.no), and was further transcribed using the MEGAshortscript T7 Transcription Kit (Ambion) and purified with the MEGAclear Transcription Clean-Up Kit (Ambion). Cas9 (GeneArt CRISPR Nuclease mRNA) was purchased from Thermo Fisher Scientific. Single cell-stage, fertilized wild-type embryos of the AB line were injected with 100 pg of *kif2a* sgRNA and 150 pg of Cas9 mRNA (in 1 nl volume). The mutation at the target site was verified via T7 endonuclease assay. The remaining sgRNA/Cas9-injected embryos were raised till adulthood, outcrossed with wild-type adults, and screened for indels by Sanger sequencing. F0 founder with germline transmission and a high rate of indels was selected to establish the knock-out line. F1 generation embryos of F0 founder were raised to adulthood, fin clipped, and sequenced. Individuals carrying 4 bp deletion of CCAG were identified and pooled together. Experiments were performed on embryos coming from homozygous and heterozygous F2 or F3 progeny.

To confirm the genotype of the larvae, a fin clip was placed in separate tubes with 50 μl of lysis buffer (100 μm Tris, 10 μm EDTA, 0.7 mm proteinase K, 0.2% Triton X-100 in Milli-Q water) to extract genomic DNA. Lysis was performed at 55°C for 3 h, followed by 10 min at 95°C. Lysed samples were genotyped by performing a PCR to amplify the genomic region spanning 4 bp deletion using KAPA HiFi HotStart Polymerase (Roche) and *kif2a*-specific primers. Successfully amplified PCR products were purified using QIAquick PCR Purification Kit (Qiagen), and the sequence was verified with Sanger sequencing (LGC Genomics). The genotypes of the individual larvae were analyzed using SeqMan software (DNASTAR Lasergene).

### Survival assay

From 1 to 5 d postfertilization (dpf) zebrafish embryos were cultured in Danieau’s medium in a 10 cm Petri dish. At 6 dpf, they were transferred to a specialized infant incubator system at 28.5°C on a 14:10 h light/dark cycle. They were fed three times a day with 1 dpf brine shrimp and dry granular food (SDS). Larvae were checked every day to a juvenile stage (30 dpf). Dead or moribund zebrafish larvae were registered, removed from the system, and frozen until further analysis. After a period of 30 d, larvae were genotyped as described previously, and survival curves were generated using a Kaplan–Meier estimate.

### Whole-mount RNA *in situ* hybridization

The 973 bp coding sequence fragment of *kif2a* was amplified from cDNA of AB wild-type strain and cloned into a Zero Blunt TOPO PCR Cloning Kit (Thermo Fisher Scientific). Cloned DNA was linearized by XhoI and HindIII, then synthesized by SP6 RNA polymerase and T7 RNA polymerase using DIG RNA labeling kit (all Roche) for sense- and antisense DIG-labeled RNA probes, respectively.

Embryos were fixed in 4% PFA, then washed with 1× PBS with Tween 20 (1× PBST), sequentially washed with 100–25% methanol and stored in 100% methanol at −20°C until needed. On the first day of whole-mount RNA *in situ* hybridization, embryos were washed with 50–25% methanol, followed by 1× PBST. After treatment with proteinase K (Sigma-Aldrich) according to the developing stages for permeabilization, embryos were fixed again with 4% PFA and washed by 1× PBST. Embryos were hybridized in Hyb^+^ solution with the *kif2a* RNA probes at 70°C overnight. On the second day, after serial washing with 2× SSCT (sodium citrate buffer with 0.1% Tween 20) with 50% formamide, 2× SSCT, and 0.2× SSCT at 70°C, embryos were blocked with 5% horse serum (Sigma-Aldrich) and incubated with anti-digoxigenin-AP Fab fragments (Roche) overnight at 4°C. On the third day, embryos were developed with BCIP/NBT (nitroblue tetrazolium) substrate (Roche). Staining was developed and stopped before the background signals started to appear in the embryos hybridized with the sense RNA probe.

### RNA extraction and quantitative RT-PCR analysis

Total RNA was extracted using TRIzol reagent (Thermo Fisher Scientific), followed by phenol-chloroform extraction, isopropanol precipitation, and ethanol washes. The resulting RNA pellet was air dried, dissolved in nuclease-free water (Thermo Fisher Scientific), and subsequently treated with RNase-free DNase (Roche). Then, 1 μg of total RNA was reverse transcribed with the High Capacity cDNA Reverse Transcription Kit (Thermo Fisher Scientific) according to the manufacturer protocol. Next, the generated cDNA was diluted (1:20) and amplified using *kif2a*- (forward, 5′-GATCACTATTCCAAGTAAA-3′; reverse, 5′-CCACTTCCTGTTTGACCATA-3′) and *kif2c*-specific primers (forward, 5′-CAAGAAGAATGACCACGCGT-3′; reverse, 5′-AGTCTCCTCTGGTAGCCTGA-3′) and 2× SsoAdvanced Universal SYBR Green Supermix (Bio-Rad) in Hard-Shell Low Profile Thin-Wall 96-well skirted PCR plates (Bio-Rad) on a CFX96 touch RT-PCR detection system (Bio-Rad) under cycling conditions according to the manufacturer protocol. The relative expression levels were quantified using the comparative Cq method (ΔΔCq) with CFX Maestro software (Bio-Rad). *kif2a* and *kif2c* transcripts were normalized against β-actin using specific primers (forward, 5′-TACAATGAGCTCCGTGTTGC-3′; reverse, 5′-TACAATGAGCTCCGTGTTGC-3′; and forward, 5’CAACAACCTGCTGGGCAAA-3′; reverse, 5′-GCGTCGATGTCGAAGGTCA-3′, respectively).

### Western blotting

Zebrafish larvae of 1–7 dpf were pooled (15 embryos for 1 dpf and 10 larvae for 2–7 dpf; all viable at the time of collection) and lysed in RIPA buffer (Sigma-Aldrich). Following the BCA assay, 30 μg of total protein was loaded onto 4–12% Bis-Tris mini gels (Thermo Fisher Scientific), separated by electrophoresis according to their molecular weight and electroblotted on nitrocellulose membranes (Thermo Fisher Scientific). Membranes were blocked in Intercept blocking buffer (LI-COR) for an hour at room temperature (RT), then incubated overnight with anti-KIF2A polyclonal rabbit antibody (catalog # ab71160, Abcam) or anti-GAPDH polyclonal antibody (catalog #SAB2701826, Sigma-Aldrich) at 4°C and subsequently with a DyLight 800 4× polyethylene glycol-conjugated secondary antibody (catalog #SA5-35571, Thermo Fisher Scientific) for 1 h at RT. Afterward, membranes were washed three times with 1× TBST for 15 min. The proteins were visualized by a biomolecular imaging system (Typhoon NIR, GE Healthcare) and bands quantified with Image Studio Lite software.

### Behavioral studies

Locomotor studies were performed as published previously (Scheldeman et al., 2017; Siekierska et al., 2019). Five to eight days postfertilization zebrafish larvae were individually arrayed in a 24-well plate containing 400 μl of Danieau’s medium per well, followed by a 20 min habituation period in an automated tracking device (Zebrabox, Viewpoint). The average total locomotor activity was quantified during a 5 min light and 10 min dark period using ZebraLab software (Viewpoint) and expressed in actinteg units, defined as the sum of all image pixel changes detected during the time of the tracking experiment. Every day, before the experiment, larvae were replenished with 400 μl of fresh Danieau’s medium. The results consist of data from quadruple experiments.

PTZ experiments were executed as published previously (Afrikanova et al., 2013). Larvae were individually arrayed in a 24-well plate containing 400 μl of Danieau’s medium per well. Subsequently, an equal volume of 12 mm PTZ (Sigma-Aldrich) was added before recording to obtain a 6 mm final PTZ concentration. The average total locomotor activity was quantified during a 30 min period using ZebraLab software (Viewpoint) and expressed in actinteg units. The results consist of data from quadruple experiments.

For habituation assay, a previously published protocol was adapted (Wolman et al., 2011). Six days postfertilization larvae were individually arrayed in a 96-well plate containing 100 μl of Danieau’s medium per well and equilibrated for 3 h in the uniformly illuminated automated tracking device (Zebrabox, Viewpoint). Subsequently, the larvae were subjected to a spaced training consisting of 120 dark flashes (DFs) with 15 s interstimulus intervals (ISIs) alternated with 10 min light periods. After a 30 min light pause, the larvae were exposed to the test assay consisting of 10 DFs with 60 s ISIs. Total movement was expressed in actinteg units. The analysis of the habituation assay was performed as previously described by Nelson et al. (2020). Spaced training results were presented as a percentage of habituation using a formula 1 – (average movement from last 30 DFs/average movement from first 30 DFs) * 100 per larva. Test results were presented as a habituation ratio of the average movement between 10 DFs from the test and the last 10 DFs from the spaced training. The results consist of data from three experiments.

### Electrophysiological studies

Noninvasive local field recordings were performed as published previously (Scheldeman et al., 2017; Siekierska et al., 2019). A 5 dpf larva was immobilized in 2% low-melting point agarose (Thermo Fisher Scientific), and the electric signal was recorded using a glass electrode that was placed on the skin of the head of a larva above the optic tectum, connected to a high-impedance amplifier filled with artificial CSF (124 mm NaCl, 2 mm KCl, 2 mm MgSO_4,_ 2 mm CaCl_2_, 1.25 mm KH_2_PO_4_, 26 mm NaHCO_3_, and 10 mm glucose). A grounding electrode and a reference electrode were placed in the recording space. The differential signal between the recording electrode and the reference electrode was amplified 10,000 times by a DAGAN 2400 Amplifier, band pass filtered at 0.3–300 Hz, and digitized at 2 kHz via a PCI-6251 interface (National Instruments) with WinEDR (John Dempster, University of Strathclyde, Glasgow, UK). Single recordings were performed for 10 min. Spontaneous electrical discharges were classified as positive events when their amplitude was at least three times the amplitude of the baseline and lasted for at least 100 ms. Analysis of the recordings was performed in a blinded manner using Clampfit 10.2 software (Molecular Devices).

PTZ experiments were executed as described previously (Afrikanova et al., 2013), larvae were arrayed in a 96-well plate containing 100 μl of Danieau’s medium. Subsequently, an equal volume of 12 mm PTZ (Sigma-Aldrich) was added for 15 min before recording to obtain a 6 mm final PTZ concentration. Electrophysiological recordings were performed and analyzed as described above. Data were expressed as the mean ± SD.

### Head and body surface area measurements

For head area measurements, larvae were positioned in 3% methylcellulose. Lateral images of 3 and 5 dpf larvae were acquired with a microscope (model MZ 10F, Leica; with a DFC310 FX digital camera, Leica) and Leica Application Suite version 3.6 software. Measurements were performed manually in ImageJ software in a blind manner. Zebrafish body surface area was measured by encircling the total surface of a larva, starting from the anterior tip of the snout to the base of the posterior caudal fin. Head surface areas were measured by tracing the boundary of the surface of interest using reference spots on the head such as the otic vesicle or dorsal indentation just above the eye, at the level of the pineal gland, as previously published (Scheldeman et al., 2017; Siekierska et al., 2019). The absolute values of measured head surface area were normalized to the body surface area of the larvae.

### Hematoxylin and eosin staining

The 5 dpf larvae were fixed in 4% PFA at 4°C overnight and kept in 70% ethanol. At least four larvae per genotype group were embedded in 1% agarose in 1× TAE buffer. A mold, specifically designed to align zebrafish larvae, was used to produce agarose blocks with identical distributed wells of the same depth. Agarose blocks were gradually dehydrated in an enclosed automated tissue processor (Shandon Excelsior ES, Thermo Fisher Scientific) and subsequently embedded in paraffin. The heads of paraffin-embedded larvae were sectioned on an HM 325 manual rotary microtome (Thermo Fisher Scientific) at a thickness of 5 μm. The specimens were stained with hematoxylin and eosin (H&E) stain using Varistain Gemini ES Automated Slide Stainer (Thermo Fisher Scientific) according to laboratory protocols. The resulting sections were imaged at 20× and 40× magnification in SPOT 5.1 software (SPOT Imaging) by a SPOT-RT3 camera mounted on a Leica microscope. The brightness of the images was adjusted for the white background. The number of sections was counted per larva per genotype and expressed as the mean ± SD. Additional counting of brain nuclei was performed to support size difference. Four equivalent sections were selected for each brain region per genotype, and hematoxylin-positive stained nuclei were counted using QuPath (0.2.3) software. The results were expressed as the average of hematoxylin-positive stained nuclei within each selected brain area.

### Whole-mount BrdU labeling

Whole-mount BrdU staining was performed on 5 dpf larvae as described previously (Tiraboschi et al., 2020). Larvae were incubated in a 10 mm BrdU (Sigma-Aldrich): 1× E3 with 15% DMSO solution for 20 min on ice. Embryos were placed at 28°C for 5 min and subsequently fixed for 2 h in 4% PFA at RT. Fixed larvae were washed three times for 5 min in 0.1% PBS-Tween at RT, incubated in 150 mm Tris-HCl, pH 9, for 15 min at RT, then at 70°C for 15 min. Larvae were washed three times for 5 min in 0.1% PBS-Tween and permeabilized in acetone for 30 min at −20°C. Afterward, larvae were incubated in 2N HCl for 1 h at RT to expose the BrdU epitope. Next, HCl was neutralized with 0.1 m borate buffer, pH 8.5, for 20 min at RT, after which larvae were washed 3× for 5 min in 0.1% PBS-Tween and afterward, again for 15 min. Larvae were bathed overnight in blocking buffer at 4°C in 0.1% PBS-Tween/10% goat serum/0.8% Triton X-100/1% BSA on a rotary shaker. The day after, blocking buffer was exchanged for BrdU antibody (clone 3D4; dilution, 1:200; BD Biosciences) in 0.1% PBS-Tween/1% goat serum/0.8% Triton X-100/1% BSA for 72 h at 4°C. Further, larvae were washed 3× for 5 min in 0.1% PBS-Tween and incubated for 5 h in 0.1% PBS-Tween/0.8% Triton X-100. Afterward, secondary antibody (dilution, 1:2000; Alexa Fluor 594 goat-anti-mouse, Abcam) and NucRed (dilution, 1:5000; Live 647 ReadyProbes Reagent, Thermo Fisher Scientific) were applied in 0.1% PBS-Tween/1% goat serum/0.8% Triton X-100/1% BSA for 72 h at 4°C. After 72 h, larvae were washed 3× for 5 min in 0.1% PBS-Tween and incubated overnight in 0.1% PBS-Tween/0.8% Triton X-100. The day after, larvae were mounted on glass slides and dorsal *z*-stacks of the optic tectum were acquired using an LSM 780–SP Mai Tai HP DS confocal microscope (Zeiss) equipped with an LD LCI Plan Apo 25×/0.8 objective. To quantify proliferative cells in the optic tectum using ImageJ, a nonspecific signal was removed by creating a mask on the nuclear dye channel. Then, a threshold was set in the BrdU channel to identify individual proliferative cells, and particle analysis was performed on a *z*-projection of this signal.

### Caspase-3 immunohistochemistry

Immunohistochemical detection of cell death was conducted on 5-μm-thick deparaffinized and rehydrated sections corresponding to the sections stained with H&E, as published previously (Siekierska et al., 2019). Specimens were subjected to heat-induced antigen retrieval by incubation in 10 mm sodium citrate, pH 6.0, for 10 min at 98°C in a water bath, followed by a 30 min cool down. Subsequently, specimens were treated twice for 8 min with 3% hydrogen peroxide. The sections were blocked for 30 min in 5% normal goat serum in 1× TBST at RT, and further incubated with primary antibody against active caspase-3 (1:500 dilution; catalog #ab13847, Abcam) for 1 h at RT. After rinsing 3× for 15 min with 1× TBST, an HRP-conjugated secondary antibody (1:200 dilution; catalog #111–035-003, Jackson ImmunoResearch) was applied for 1 h at RT. The slides were treated with DAB^+^/chromogen (DAKO) for 1 min at RT and rinsed with deionized water. As a nuclear counterstain, hematoxylin was added for 3 min and the specimens were subsequently rinsed in deionized water. After clearing in ethanol and histoclear, the slides were mounted. For each staining, a negative control was included by processing sections in the absence of the primary antibody. The images were taken at 20× magnification in a SPOT 5.1 software (SPOT Imaging) by a SPOT-RT3 camera mounted on a Leica microscope. The brightness of the images was adjusted for the white background. The number of DAB positively stained nuclei in the whole brain was counted using QuPath (0.2.3) software per larva per genotype. The results were expressed as the percentage of apoptotic cells ± SD (DAB positively stained nuclei over the total cell number) per larva per genotype.

### Experimental design and statistical analysis

When comparing parametric data from two groups, an unpaired Student’s *t* test was used. If the data were from more than two groups, a one-way ANOVA with a Dunnett’s multiple-comparisons test were used (Table 1). Outliers were identified from datasets using the ROUT method (*Q* = 1%). The significance of all statistical comparisons was set at *p* < 0.05. All statistical comparisons were performed using GraphPad Prism (version 8.0.0) software.

## Results

### Mutation of the *kif2a* gene by CRISPR/Cas9 results in a stable homozygous mutant zebrafish line

**Figure 1.**
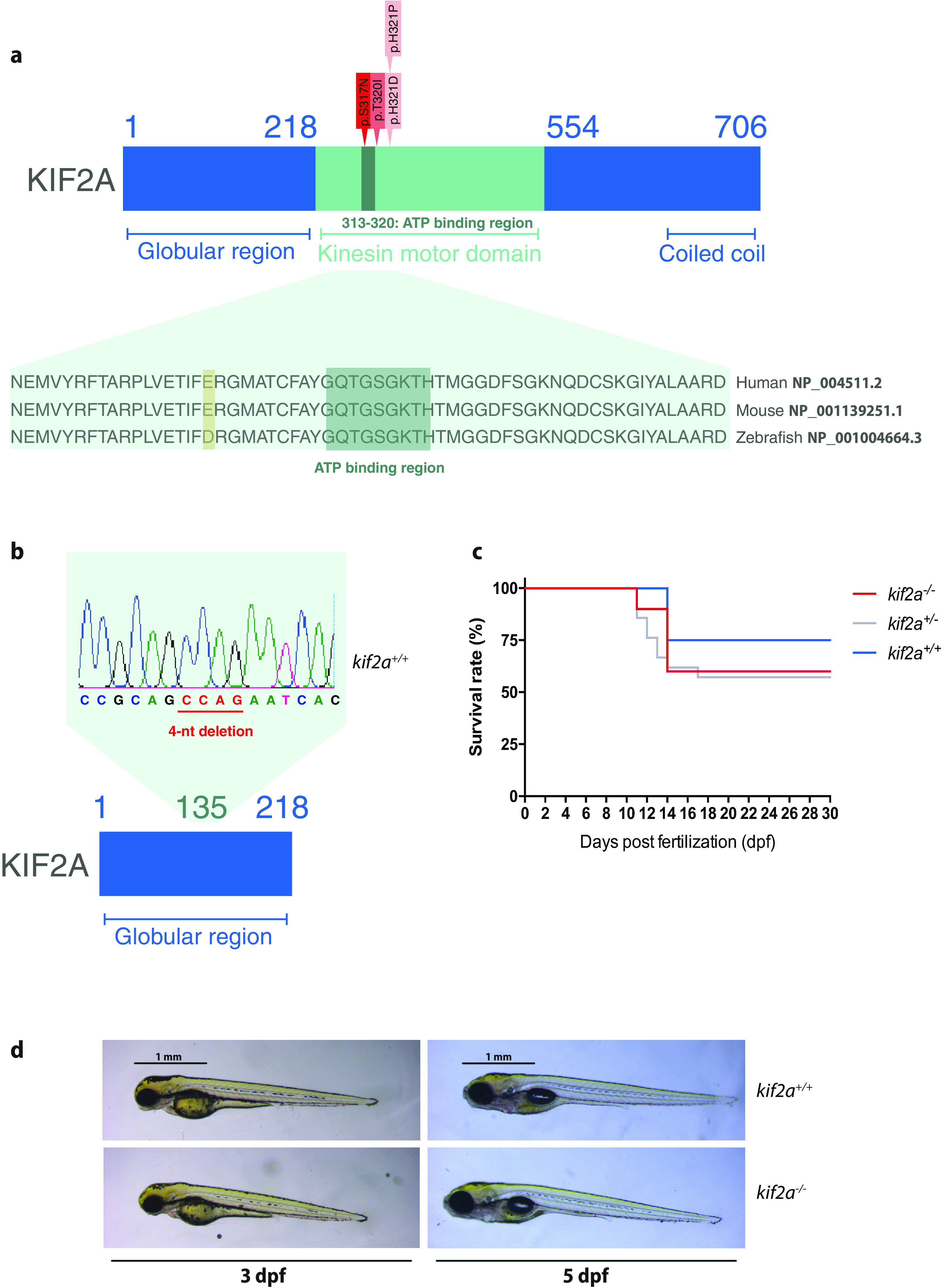
A novel *kif2a* knock-out zebrafish model shows that *kif2a* is not indispensable for survival and mutations do not result in gross morphologic abnormalities. ***a***, Linear representation of the human kinesin 2A polypeptide with highlighted four *de novo* MCD-associated mutations (indicated in red-pink) located in or near the ATP-binding region (indicated in green). Kinesin motor domain, coiled coil, and globular domains (UniProtKB-O00139) are highlighted. Multiple sequence alignment shows the level of conservation of the ATP binding region (indicated in dark turquoise) of human, mouse, and zebrafish. ***b***, Localization of 4-nucleotide deletion (CCAG) in the globular region of Kif2a and electropherogram of *kif2a^+/+^* larvae, confirming the presence of the mutation in homozygotes. ***c***, Kaplan–Meier survival rate curves of *kif2a^+/+^* (*n* = 5), *kif2a*^+/−^ (*n* = 21), and *kif2a*^−/−^ (*n* = 10) larvae. Larvae were obtained by the mating of heterozygous adults. Surviving larvae were counted every 24 h until 30 dpf. ***d***, Lateral bright-field images representing normal macroscopic morphology of 3 and 5 dpf *kif2a^+/+^* and *kif2a*^−/−^ larvae. Scale bar, 1 mm. The design of the CRISPR/Cas9 *kif2a* zebrafish knock-out line is illustrated in Extended Data Figure 1-1. qPCR analysis of *kif2c* expression levels showed no upregulation of *kif2c* during development in *kif2a*^−/−^ larvae (Extended Data Fig. 1-1).

Zebrafish genome encodes a single *KIF2A* ortholog that shows a high degree of conservation between mouse and human proteins (specifically within the kinesin motor domain), with 84.66% identity and 89% similarity for human and zebrafish (Fig. 1*a*). To investigate *KIF2A* loss of function *in vivo*, a zebrafish knock-out model was generated by the CRISPR/Cas9 genome-editing technology (Hruscha et al., 2013; Hwang et al., 2013). We targeted the fifth exon of the zebrafish *kif2a* located in the globular N-terminal domain. A positive founder, transmitting a premature stop codon, was ultimately selected and used to obtain the F2 generation (Extended Data Fig. 1-1*a*,*b*). Genomic analysis confirmed the presence of the mutation in *kif2a*^−/−^ larvae (Fig. 1*b*).

**Table 1:** Statistical table

Figure	Data structure	Type of test	Power
1*c*	NA	Log-rank Mantel–Cox	0.05 (95% confidence interval)
1–1*c*	Gaussian distribution	Unpaired Student’s *t* test	0.05 (95% confidence interval)
1–1*d*	Gaussian distribution	Unpaired Student’s *t* test	0.05 (95% confidence interval)
2*a*	Gaussian distribution	One-way ANOVA with Dunnett’s multiple-comparisons test	0.05 (95% confidence interval)
2*b*	Gaussian distribution	One-way ANOVA with Dunnett’s multiple-comparisons test	0.05 (95% confidence interval)
3*a*	Gaussian distribution	Unpaired Student’s *t* test per day	0.05 (95% confidence interval)
3*b*	Gaussian distribution	Unpaired Student’s *t* test	0.05 (95% confidence interval)
3*c*	Gaussian distribution	Unpaired Student’s *t* test	0.05 (95% confidence interval)
3*d*	Gaussian distribution	Unpaired Student’s *t* test	0.05 (95% confidence interval)
3–1*a*	Gaussian distribution	One-way ANOVA with Dunnett’s multiple-comparisons test per day	0.05 (95% confidence interval)
3–1*b*	Gaussian distribution	One-way ANOVA with Dunnett’s multiple-comparisons test	0.05 (95% confidence interval)
3–1*c*	Gaussian distribution	One-way ANOVA with Dunnett’s multiple-comparisons test	0.05 (95% confidence interval)
4*b*	Gaussian distribution	Unpaired Student’s *t* test	0.05 (95% confidence interval)
4*c*	Gaussian distribution	Unpaired Student’s *t* test	0.05 (95% confidence interval)
4–1*a*	Gaussian distribution	One-way ANOVA with Dunnett’s multiple-comparisons test	0.05 (95% confidence interval)
4–1*b*	Gaussian distribution	One-way ANOVA with Dunnett’s multiple-comparisons test	0.05 (95% confidence interval)
5*a–d*	Gaussian distribution	Unpaired Student’s *t* test	0.05 (95% confidence interval)
5*f*	Gaussian distribution	Unpaired Student’s *t* test	0.05 (95% confidence interval)
5*g,h*	Gaussian distribution	Unpaired Student’s *t* test	0.05 (95% confidence interval)
5–1*b*	Gaussian distribution	Unpaired Student’s *t* test	0.05 (95% confidence interval)
5–1*c–h*	Gaussian distribution	Unpaired Student’s *t* test	0.05 (95% confidence interval)
6*b*	Gaussian distribution	Unpaired Student’s *t* test	0.05 (95% confidence interval)
6*e*	Gaussian distribution	Unpaired Student’s *t* test	0.05 (95% confidence interval)
6*h*	Gaussian distribution	Unpaired Student’s *t* test	0.05 (95% confidence interval)

10.1523/ENEURO.0055-21.2021.f1-1 Figure 1-1 Design of a CRISPR/Cas9 *kif2a* zebrafish knock-out line. ***a***, Scheme illustrating the generation process of *kif2a* CRISPR line. Single cell-stage wild type (WT) AB embryos were injected with *kif2a* sgRNA and Cas9 mRNA. Injected embryos were raised until adulthood, and among them an F0 founder was selected that was outcrossed with WT zebrafish. F1 generation embryos were raised till adulthood, fin clipped, and genotyped via Sanger sequencing. Zebrafish carrying the identical mutation (4 bp deletion) were identified and pooled together. ***b***, Ensembl image of the *kif2a* zebrafish gene structure (ENSDARG00000043571) showing its 20 exons and mutated exon 5 affecting the globular N-terminal domain. ***c***, qPCR analysis of expression levels of *kif2c* in 2 dpf *kif2a^+/+^* and *kif2a*^−/−^ larvae. Data are represented as fold change expression (10 heads per sample used). ***d***, qPCR analysis of expression levels of *kif2c* in 5 dpf *kif2a^+/+^* and *kif2a*^−/−^ larvae. Data are represented as fold change expression (seven heads per sample used). Download Figure 1-1, EPS file.

### *kif2a*^−/−^ larvae survive until adulthood and are fertile

Homozygous larvae obtained by the mating of heterozygous adults segregated according to a Mendelian ratio (1:2:1). Surprisingly, *kif2a*^−/−^ larvae were able to survive until adulthood. Kaplan–Meier curves showed that at 30 dpf the recorded survival rate was 75% for *kif2a^+/+^* (*n* = 5), 60% for *kif2a*^−/−^ (*n* = 10), and 57% for *kif2a*^+/−^ (*n* = 21) larvae (*χ^2^* = 0.65, *p *=* *0.72, log-rank Mantel–Cox test; Fig. 1*c*). Moreover, the offspring of incrossed homozygotes was viable and able to be raised until adulthood, and these homozygous progenies were used to perform the experiments described in this study, unless differently specified.

Visual inspection of the development of *kif2a^+/+^* and *kif2a*^−/−^ larvae from 3 until 5 dpf was performed. No obvious macroscopic differences in appearance such as gross deformation, edema, necrosis, or impairment in touch response were detected, making *kif2a*^−/−^ larvae morphologically inconspicuous when compared with *kif2a^+/+^* animals (Fig. 1*d*). The same trend was observed for adult mutants, which were all morphologically similar to their *kif2a^+/+^* and *kif2a*^+/−^ siblings (data not shown).

Since *kif2a*^−/−^ larvae were viable and morphologically indistinguishable from their *kif2a^+/+^* siblings, we investigated whether the loss of Kif2a might result in a direct genetic compensation from another member of *kinesin-13* family, *kif2c*. Quantitative PCR (qPCR) results did not show any increase in *kif2c* expression levels in *kif2a*^−/−^ larvae compared with their *kif2a^+/+^* siblings either at 2 dpf (*kif2a*^−/−^: 1.142 ± 0.3583, *n* = 5; *kif2a^+/+^*: 1.0 ± 0.0, *n* = 5, Student’s *t* test; Extended Data Fig. 1-1*c*) or at 5 dpf (*kif2a*^−/−^: 1.098 ± 0.2887, *n* = 2; *kif2a^+/+^*: 1.0 ± 0.0, *n* = 2, Student’s *t* test; Extended Data Fig. 1-1*d*; i.e., at time points of peak and declining levels of *kif2a* RNA and Kif2a protein expression, respectively) observed in *kif2a^+/+^* larvae.

### Temporal and spatial expression patterns reveal a loss of *kif2a* mRNA in the mutant line

**Figure 2.**
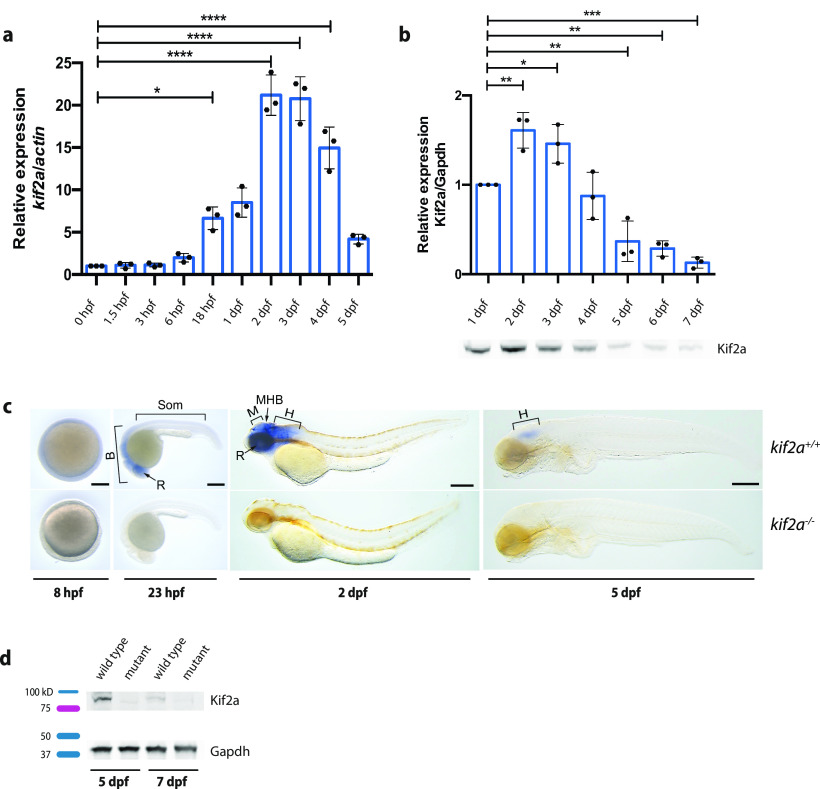
Expression and localization pattern of *kif2a* in the developing zebrafish. ***a***, qPCR analysis of *kif2a* levels in *kif2a^+/+^* larvae normalized to actin and represented as the fold change expression to 0 hpf. Values are reported as the mean ± SEM of three separate experiments. Significant values are noted as ****p ≤ 0.0001 and *p ≤ 0.05. ***b***, Relative quantification of Kif2a protein expression in *kif2a*^+/+^ larvae of 1–7 dpf normalized to Gapdh and represented as the fold change expression to 1 dpf. Values are reported as the mean ± SEM of three separate experiments. Significant values are noted as ***p ≤ 0.001, **p ≤ 0.01, and *p ≤ 0.05. Below the graph is a representative Western blot image of Kif2a protein expression levels in *kif2a*^+/+^ larvae of 1–7 dpf. ***c***, Spatiotemporal expression patterns of *kif2a* by whole-mount RNA *in situ* hybridization at 8 hpf, 23 hpf, 2 dpf, and 5 dpf. B, Brain; H, hindbrain; M, midbrain; MBH, midbrain–hindbrain boundary; Som, somite; R, retina. Scale bar, 200 μm. ***d***, Representative Western blot image of Kif2a protein expression levels comparing *kif2a*^+/+^ with *kif2a*^−/−^ larvae at 5 and 7 dpf.

We investigated the expression levels of *kif2a* RNA and Kif2a protein during 1–7 dpf. Both *kif2a* transcript (Fig. 2*a*) and Kif2a protein (Fig. 2*b*) expression were found to increase during the first days of development, with peak expression levels at 2 dpf (transcript: 2119 ± 238.2, *n* = 3; protein: 1.609 ± 0.1993, *n* = 3) and 3 dpf (transcript: 2077 ± 259.8, *n* = 3; protein: 1.459 ± 0.2164, *n* = 3). At 5 dpf (transcript: 418.8 ± 57.25, *n* = 3; protein: 0.3680 ± 0.2259, *n* = 3), however, the expression decreased, with an almost complete absence of protein at 7 dpf (0.1299 ± 0.06,029, *n* = 3; *p *<* *0.0001, ANOVA, Dunnett’s multiple-comparisons test; Fig. 2*a*,*b*).

To understand the potential function of *kif2a* during development, its spatial expression patterns in *kif2a*^+/+^ and *kif2a*^−/−^ larvae were examined. The 8 h postfertilization (hpf) *kif2a* mRNA was found to be equally expressed in the whole embryo. At 23 hpf, the expression was limited to the head, and gradually became restricted to midbrain and hindbrain at 2 dpf. At 5 dpf, *kif2a* mRNA was observed only in the hindbrain (Fig. 2*c*). In *kif2a*^−/−^ larvae, no mRNA expression was detected at any developmental stage examined (Fig. 2*c*). This suggests nonsense-mediated mRNA decay (NMD) because of the mutation resulting in a premature termination codon and thus the total absence of mRNA. Additionally, we also confirmed that there was an absence of protein in *kif2a*^−/−^ larvae at 5 and 7 dpf, confirming the efficiency of the knock-out (Fig. 2*d*).

### *kif2a*^−/−^ larvae display neurobehavioral alterations like hypoactivity, habituation learning deficits, and increased locomotor behavior to PTZ

**Figure 3.**
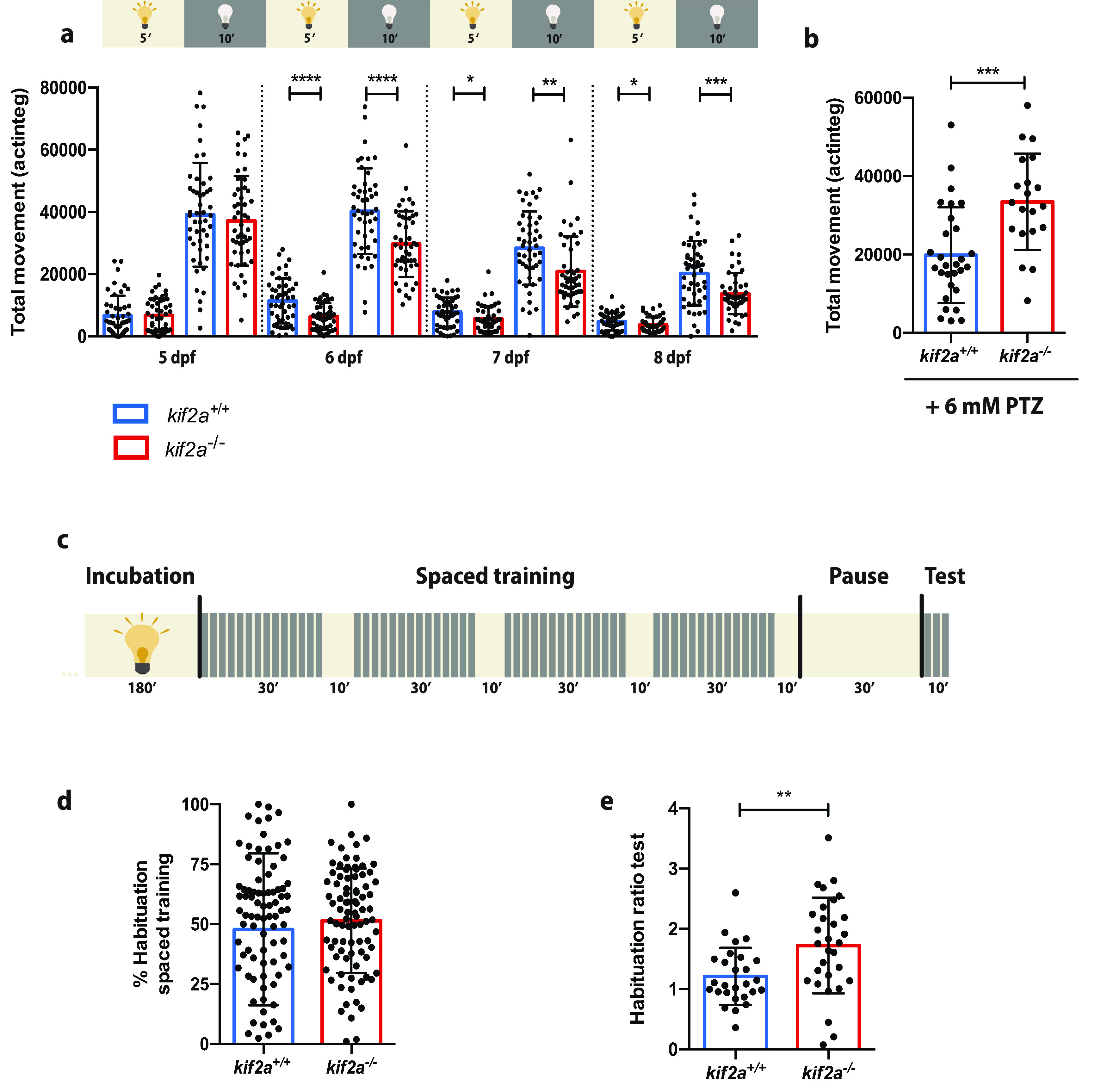
*kif2a*^−/−^ larvae exhibit locomotor abnormalities, increased locomotor behavior to PTZ and habituation learning disabilities. ***a***, Average total movement of *kif2a^+/+^* and *kif2a*^−/−^ larvae from 5 to 8 dpf expressed in actinteg units. Values are reported as the mean ± SD of four separate experiments. Significant values are noted as ****p ≤ 0.0001, ***p ≤ 0.001, **p ≤ 0.01, and *p ≤ 0.05. ***b***, Average total movement of 5 dpf *kif2a^+/+^* and *kif2a*^−/−^ larvae after 6 mm PTZ immersion for 30 min. Values are reported as the mean ± SD of four separate experiments. Significant values are noted as ***p ≤ 0.001. ***c***, The habituation learning assay performed on 6 dpf *kif2a^+/+^* and *kif2a*^−/−^ larvae was composed of different protocols: starting with a 3 h incubation period in the light (indicated in yellow), followed by a habituation training consisting of four periods with 120 DFs with a 15 s ISI (regions with gray and yellow stripes), alternated by 10 min of light, a period of 30 min pause in the light preceding the actual test, and a test consisting of 10 DFs with a 60 s ISI (region with gray and yellow stripes). ***d***, Percentage habituation of *kif2a^+/+^* and *kif2a*^−/−^ larvae to DFs during the spaced training. Values are reported as the mean ± SD of three separate experiments. ***e***, Habituation ratio of average movement of *kif2a^+/+^* and *kif2a*^−/−^ larvae to DFs during the test. Values are reported as the mean ± SD of three separate experiments. Significant values are noted as **p ≤ 0.01. No locomotor abnormalities and habituation learning disabilities were observed in *kif2a*^−/−^ zebrafish larvae from +/– incrosses (Extended Data Fig. 3-1).

To investigate the impact of Kif2a loss of function on behavioral activity, locomotor experiments were performed on 5–8 dpf *kif2a^+/+^* and *kif2a*^−/−^ larvae subjected to a light/dark stimulus. *kif2a*^−/−^ larvae showed significantly decreased swimming activity during both the light and dark phases from 6–8 dpf (6 dpf: light, 6389.083 ± 4322.520, *n* = 48, *p *<* *0.0001; dark, 29,666.960 ± 10,566.630, *n* = 48, *p *<* *0.0001; 7 dpf: light, 5642.745 ± 4051.766, *n* = 48, *p *=* *0.0194; dark, 20,825.320 ± 11,267.100, *n* = 48, *p *=* *0.0019; 8 dpf: light, 3665.851 ± 2489.406, *n* = 47, *p *=* *0.0452; dark, 13,710.230 ± 6626.408, *n* = 47, *p *=* *0.0004, Student’s *t* test; Fig. 3*a*) compared with their *kif2a^+/+^* siblings (6 dpf: light, 11,400.170 ± 7202.550, *n* = 48; dark, 40,244.920 ± 13,768.350, *n* = 48; 7 dpf: light, 7773.229 ± 4651.655, *n* = 48; dark, 28,379.650 ± 11,812.280, *n* = 48; 8 dpf: light, 4793.500 ± 2904.615, *n* = 48; dark, 20,260.730 ± 10,363.520, *n* = 48). At 5 dpf, however, no significant decrease was observed between the *kif2a*^−/−^ larvae (light: 6705.521 ± 5427.908, *n* = 48, *p *=* *0.9174; dark: 37,165.790 ± 14,433.510, *n* = 48, *p *=* *0.5394, Student’s *t* test; Fig. 3*a*) and *kif2a^+/+^* larvae (light: 6578.854 ± 6460.863, *n* = 48; dark: 39,130.670 ± 16,734.680, *n* = 48).

10.1523/ENEURO.0055-21.2021.f3-1 Figure 3-1 *kif2a*^−/−^ zebrafish larvae from +/– incrosses do not exhibit locomotor abnormalities and habituation learning disabilities as seen in larvae from –/– incrosses. ***a***, Average total movement of *kif2a^+/+^*, *kif2a*^+/–^, and *kif2a*^–/–^ larvae from 5 to 8 dpf expressed in actinteg units. Values are reported as the mean ± SD of three separate experiments. ***b***, Cognition assay performed on 6 dpf *kif2a^+/+^* and *kif2a*^−/−^ larvae. The assay was composed of different protocols: starting with a 3 h incubation period in the light (indicated in yellow), followed by a habituation training consisting of four periods with 120 DFs with a 15 s ISI (regions with gray and yellow stripes), alternated by 10 min of light, a 30 min pause in the light preceding the actual test, and a test consisting of 10 DFs with a 60 s ISI (region with gray and yellow stripes). ***c***, Percentage of habituation of *kif2a^+/+^*, *kif2a*^+/–^, and *kif2a*^–/–^ larvae to DFs during the spaced training. Values are reported as the mean ± SD of three separate experiments. ***e***, Habituation ratio of average movement of *kif2a^+/+^*, *kif2a*^+/–^, and *kif2a*^–/–^ larvae to DFs during the test. Values are reported as the mean ± SD of three separate experiments. Download Figure 3-1, EPS file.

Moreover, since we observed hypoactivity in *kif2a*^−/−^ larvae, we investigated whether they presented increased susceptibility to seizures by the administration of a chemical convulsant. *kif2a*^−/−^ animals were incubated with a subthreshold dose of PTZ, a GABA_A_ receptor antagonist that is widely used to induce acute seizures in zebrafish (Afrikanova et al., 2013). We found a significant increase in epileptiform activity in *kif2a*^−/−^ larvae (33,431 ± 12,315, *n* = 20, *p *=* *0.0004, Student’s *t* test; Fig. 3*b*) after PTZ incubation in comparison to their *kif2a^+/+^* siblings (19,807 ± 12,194, *n* = 20), confirming increased seizure susceptibility.

Additionally, to evaluate whether *kif2a*^−/−^ larvae deriving from heterozygous parents had a similar phenotype, we performed the same experiments on heterozygous offspring. Our results demonstrated that there was no difference at any day postfertilization between the swimming activity of *kif2a*^−/−^ larvae (5 dpf: light, 9282 ± 6292, *n* = 54, *p *=* *0.6908; dark, 48,504 ± 14,603, *n* = 54, *p *=* *0.7322; 6 dpf: light, 8405 ± 5250, *n* = 55, *p *=* *0.0671; dark, 39,557 ± 13,571, *n* = 55, *p *=* *0.5420; 7 dpf: light, 6258 ± 4875, *n* = 55, *p *=* *0.3670; dark, 29,524 ± 12,700, *n* = 55, *p *=* *0.8121; 8 dpf: light, 4707 ± 3545, *n* = 55, *p *=* *0.7292; dark, 19,812 ± 11,920, *n* = 55, *p *=* *0.0.2895, ANOVA, Dunnett’s multiple-comparisons test; Extended Data Fig. 3-1*a*) compared with their *kif2a^+/+^* siblings (5 dpf: light, 8208 ± 7027, *n* = 51; dark, 46,291 ± 16,613, *n* = 51; 6 dpf: light, 6242 ± 4024, *n* = 51; dark, 36,549 ± 16,230, *n* = 51; 7 dpf: light, 5757 ± 4283, *n* = 51; dark, 28,702 ± 13,452, *n* = 51; 8 dpf: light, 4329 ± 3365, *n* = 51; dark, 16,375 ± 11,071, *n* = 51). Similarly, *kif2a*^+/−^ larvae did not do not present with an abnormal behavioral phenotype (5 dpf: light, 8418 ± 7369, *n* = 117; dark, 48,354 ± 17,752, *n* = 117; 6 dpf: light, 7370 ± 4807, *n* = 117; dark, 38,840 ± 14,707, *n* = 117; 7 dpf: light, 6800 ± 4390, *n* = 116; dark, 30,264 ± 15,938, *n* = 116; 8 dpf: light, 4792 ± 3529, *n* = 117; dark, 19,030 ± 12,283, *n* = 117). Overall, these results confirm that an offspring from homozygous parents is needed to have the full-blown phenotype.

Since MCDs are an important cause of developmental delay and cognitive deficits (Desikan and Barkovich, 2016; Represa, 2019), we also examined whether *kif2a*^−/−^ larvae had difficulties in acquiring, storing, and recalling learned information by performing a habituation learning assay (Wolman et al., 2011). During the experiment, larvae were subjected to a spaced training consisting of multiple dark flashes (DFs) alternated with 10 min light periods and, after a pause, were further exposed to a test assay consisting of a block of DFs (Fig. 3*c*). *kif2a^+/+^* (47.82 ± 31.70, *n* = 87) and *kif2a*^−/−^ (51.41 ± 21.77, *n* = 88, *p *=* *0.3833, Student’s *t* test; Fig. 3*d*) larvae showed an adaptation profile similar to DFs during the habituation, as quantified by calculation of the habituation percentage. Strikingly, *kif2a*^−/−^ larvae (1.723 ± 0.7958, *n* = 30, *p *=* *0.0053, Student’s *t* test; Fig. 3*e*) demonstrated memory deficits as they were not able to respond to DFs in the same way as their *kif2a^+/+^* siblings (1.212 ± 0.4738, *n* = 27) during the training, after a period of inactivation. These results demonstrate that *kif2a* deficiency results in disability in habituation, suggesting memory decay and learning disabilities.

Correspondingly, to characterize the phenotype of *kif2a*^−/−^ larvae deriving from heterozygous parents, this experiment was performed on heterozygous offspring (Extended Data Fig. 3-1*b–d*). Both *kif2a*^+/−^ (percentage of habituation: 42.39 ± 35.83%, *n* = 66; habituation ratio: 1.161 ± 0.6025, *n* = 30) and *kif2a*^−/−^ larvae (percentage of habituation: 35.92 ± 33.74%, *n* = 39; *p* = 0.5279, ANOVA, Dunnett’s multiple-comparisons test; Extended Data Fig. 3-1*c*; habituation ratio: 1.216 ± 0.5361, *n* = 30, *p *=* *0.9423, ANOVA, Dunnett’s multiple-comparisons test; Extended Data Fig. 3-1*d*) showed a habituation and learning profile similar to that of their *kif2a^+/+^* siblings (percentage of habituation: 35.76 ± 39.16, *n* = 22; habituation ratio: 1.205 ± 0.7903, *n* = 29), confirming that an offspring from homozygous adults is needed to obtain a full-blown phenotype.

### *kif2a*^−/−^ larvae present increased seizure susceptibility to PTZ

**Figure 4.**
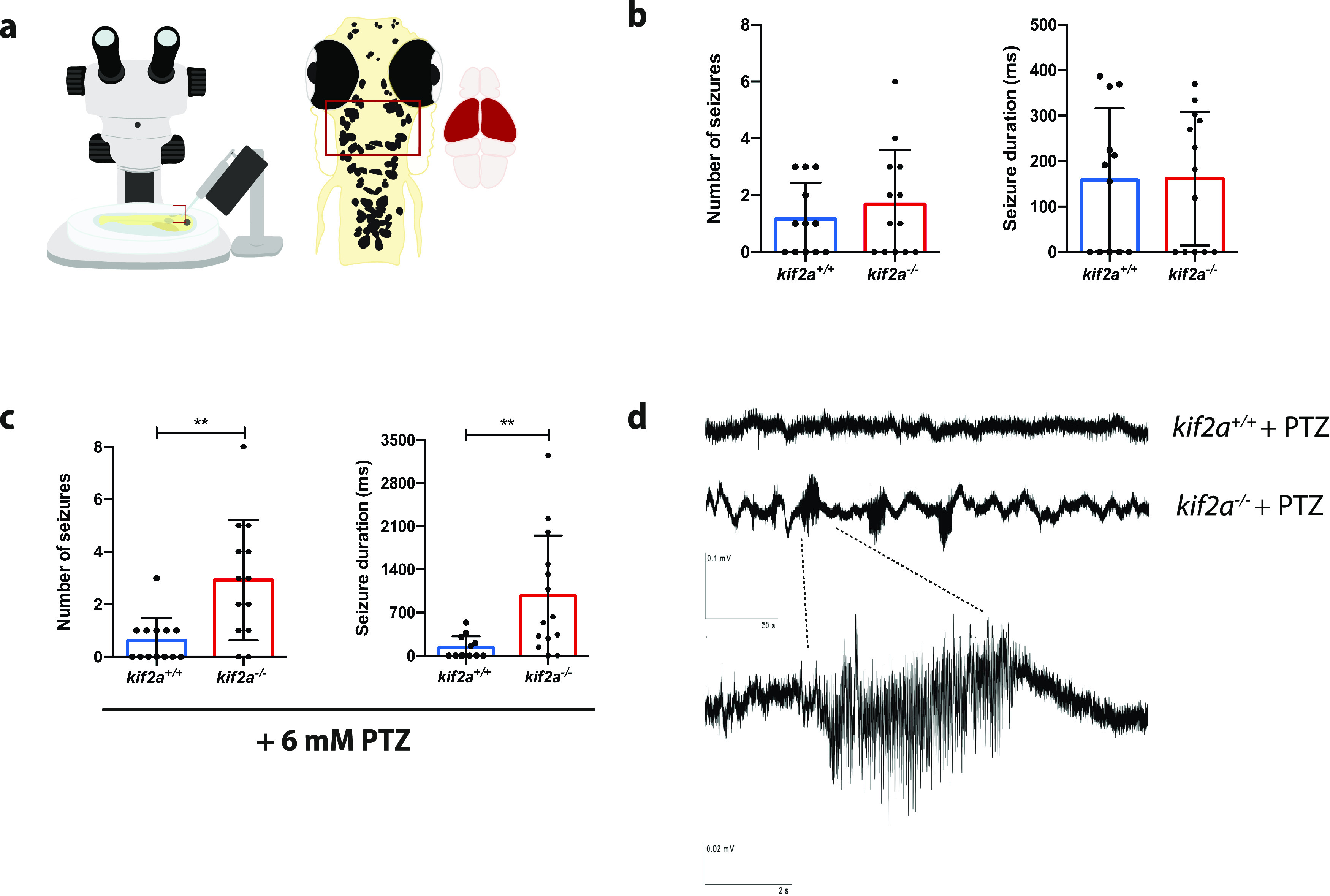
*kif2a*^−/−^ larvae show increased susceptibility to epilepsy. ***a***, Visual representation of the setup of local field potential recording from optic tectum (indicated in red) of 5 dpf larvae. ***b***, Number of seizures and average seizure duration in *kif2a^+/+^* and *kif2a*^−/−^ larvae. ***c***, Number of seizures and average seizure duration in *kif2a^+/+^* and *kif2a*^−/−^ larvae after 6 mm PTZ immersion for 15 min. ***d***, Representative fragments of a 10 min recording (calibration: 0.1 mV, 20 s) of *kif2a^+/+^* and *kif2a*^−/−^ larvae after 6 mm PTZ immersion for 15 min, with enlargement of a polyspiking event (calibration: 0.02 mV, 2 s) in *kif2a*^−/−^ larvae. Data are represented as the mean ± SD. Significant values are noted as **p ≤ 0.01. No increased seizure susceptibility was observed in the brains of *kif2a*^−/−^ zebrafish larvae from +/– incrosses (Extended Data Fig. 4-1).

To investigate whether *kif2a* knockout resulted in abnormal epileptic brain activity, noninvasive local field potential recordings were performed from the optic tecta of 5 dpf *kif2a^+/+^* and *kif2a*^−/−^ larvae (Fig. 4*a*). Events were classified as epileptiform if the amplitude equaled or exceeded three times the baseline. No significant increase in abnormal spontaneous epileptiform activity was detected in *kif2a*^−/−^ larvae (seizure number: 1.692 ± 1.888, *n* = 13, *p *=* *0.4262, Student’s *t* test; Fig. 4*b*, left; seizure duration: 430.0 ms ± 494.3, *n* = 13, *p *=* *0.0692, Student’s *t* test; Fig. 4*b*, right) compared with *kif2a^+/+^* larvae (seizure number: 1.167 ± 1.267, *n* = 12; seizure duration: 104.2 ms ± 127.6, *n* = 9). We then tried to determine whether *kif2a*^−/−^ larvae were presenting increased susceptibility to seizures by the administration of a convulsant. *kif2a*^−/−^ animals were incubated with a subthreshold dose of PTZ. We found a significant increase in epileptiform activity in *kif2a*^−/−^ larvae after PTZ incubation (seizure number: 2.923 ± 2.29, *n* = 13, *p *=* *0.0024, Student’s *t* test; Fig. 4*c*, left; seizure duration: 2360 ms ± 2723, *n* = 12, *p *=* *0.0120, Student’s *t* test; Fig. 4*c*, right) compared with their *kif2a^+/+^* siblings (seizure number: 0.6154 ± 0.8697, *n* = 13; seizure duration: 94.59 ms ± 141.5, *n* = 11), confirming increased seizure susceptibility (Fig. 4*c*,*d*).

10.1523/ENEURO.0055-21.2021.f4-1 Figure 4-1 Brains of *kif2a*^−/−^ zebrafish larvae from +/– incrosses do not show increased susceptibility to epilepsy as seen in brains of larvae from −/− incrosses. ***a***, Number of seizures and average seizure duration in *kif2a^+/+^*, *kif2a*^+/–^, and *kif2a*^−/−^ larvae. ***b***, Number of seizures and average seizure duration in *kif2a^+/+^*, *kif2a*^+/–^, and *kif2a*^−/−^ larvae after 6 mM PTZ immersion for 15 min. Download Figure 4-1, EPS file.

Again, we evaluated whether *kif2a*^−/−^ larvae deriving from heterozygous parents presented with spontaneous abnormal epileptic brain activity or increased PTZ seizure susceptibility. Neither *kif2a*^+/−^ (seizure number: 0.5 ± 0.6325, *n* = 16, *p *=* *0.1953; seizure duration: 114.0 ms ± 148.7, *n* = 17) and *kif2a*^−/−^ larvae (seizure number: 0.0 ms ± 0.0, *n* = 7, *p *= 0.1953, ANOVA, Dunnett’s multiple-comparisons test; Extended Data Fig. 4-1*a*, left; seizure duration: 0.0 ± 0.0, *n* = 7, *p *=* *0.0759, ANOVA, Dunnett’s multiple-comparisons test; Extended Data Fig. 4-1*a*, right) showed any spontaneous abnormal epileptiform brain activity compared with their *kif2a^+/+^* siblings (seizure number: 0.3750 ± 0.744, *n* = 8; seizure duration: 36.44 ms ± 73.76, *n* = 8). Similarly, no significantly increased PTZ seizure susceptibility was observed in *kif2a*^+/−^ larvae (seizure number: 1.545 ± 1.968, *n* = 11, *p *=* *0.3183; seizure duration: 63.61 ms ± 80.90, *n* = 9) and *kif2a*^−/−^ larvae (seizure number: 1.4 ± 2.271, *n* = 10, *p* = 0.3183, ANOVA, Dunnett’s multiple-comparisons test; Extended Data Fig. 4-1*b*, left; seizure duration: 160.9 ms ± 304, *n* = 9, *p *=* *0.3332, ANOVA, Dunnett’s multiple-comparisons test; Extended Data Fig. 4-1*b*, right) compared with their *kif2a^+/+^* siblings (seizure number: 0.0 ms ± 0.0, *n* = 5; seizure duration: 0.0 ms ± 0.0, *n* = 5).

### Aberrant head morphology of *kif2a* mutants is associated with defects in neurologic development

**Figure 5.**
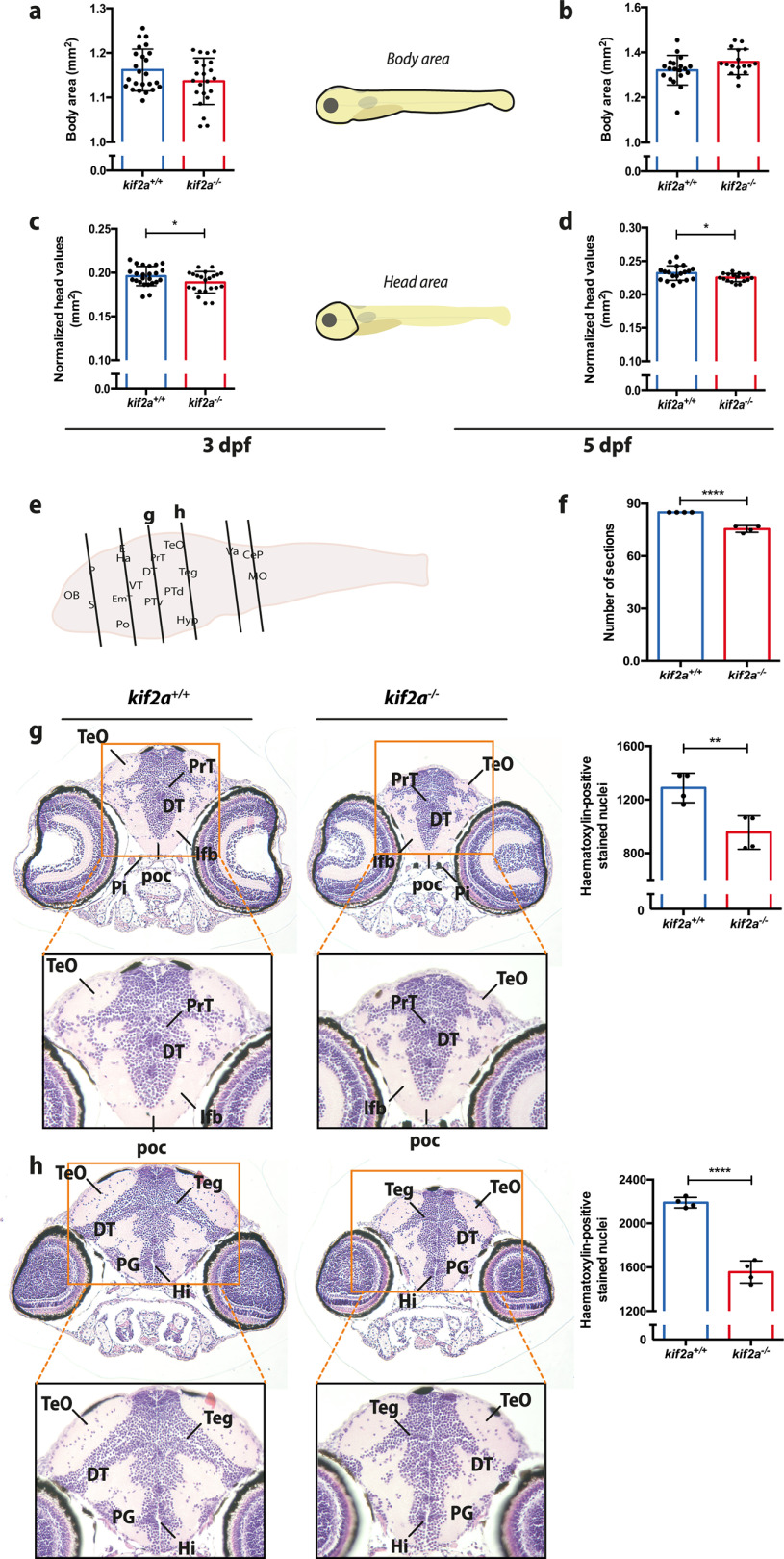
*kif2a* larvae present microcephaly that is associated with defects in neurologic development. ***a–d***, Comparison of the individual measurements for body area (***a***, ***b***) and head size (***c***, ***d***) for *kif2a^+/+^* and *kif2a*^−/−^ larvae at 3 and 5 dpf. Data are represented as the mean ± SD. Significant values are noted as *p ≤ 0.05. ***e***, Histologic assessment of 5 dpf *kif2a*^−/−^ and *kif2a^+/+^* larval brains. Six brain regions (from forebrain to hindbrain) were selected per genotype as indicated in the diagram. ***f***, Comparison of the number of brain sections from forebrain to hindbrain (***e***) between *kif2a^+/+^* and *kif2a*^−/−^ larvae. Data are represented as the mean ± SD. Significant values are noted as ****p ≤ 0.0001. ***g***, ***h***, Coronal sections stained with H&E imaged at 20× and 40× (indicated by orange square) magnification. DT, Dorsal thalamus; Hi, intermediate hypothalamus; lfb, lateral forebrain bundle; PG, preglomerular complex; PrT, pretectum; Teg, midbrain tegmentum; TeO, tectum opticum. Bar graphs compare hematoxylin-positive stained nuclei between *kif2a^+/+^* and *kif2a*^−/−^ larvae. Data are represented as the mean ± SD. Significant values are noted as ****p ≤ 0.0001 and **p ≤ 0.01. A significant neuronal loss was observed in specific brain regions of *kif2a*^−/−^ zebrafish larvae (Extended Data Fig. 5-1).

To investigate whether the loss of function of *kif2a* was associated with microcephaly, one of the clinical symptoms of patients with *KIF2A* mutations (Tian et al., 2016; Cavallin et al., 2017), head surface areas of 3 and 5 dpf *kif2a*^−/−^ larvae were measured and normalized against total body surface. At 3 dpf, a statistically significant change in head size was observed between *kif2a^+/+^* (0.1961 ± 0.01106, *n* = 24) and *kif2a*^−/−^ zebrafish larvae (0.1890 ± 0.01226, *n* = 22; *p *=* *0.0427, Student’s *t* test; Fig. 5*c*) that was also present in 5 dpf *kif2a^+/+^* (0.2321 ± 0.01094, *n* = 19) and *kif2a*^−/−^ larvae (0.2252 ± 0.006116, *n* = 17; *p *=* *0.0295, Student’s *t* test; Fig. 5*d*). Body area did not differ significantly between the larval groups at both 3 dpf (*kif2a^+/+^*: 1.162 ± 0.047, *n* = 24; *kif2a*^−/−^: 1.136 ± 0.052, *n* = 22; *p *=* *0.0899, Student’s *t* test; Fig. 5*a*) and 5 dpf (*kif2a^+/+^*: 1.321 ± 0.066, *n* = 19; *kif2a*^−/−^: 1.358 ± 0.056, *n* = 17; *p *= 0.0801, Student’s *t* test; Fig. 5*b*).

10.1523/ENEURO.0055-21.2021.f5-1 Figure 5-1 H&E histological staining of *kif2a*^−/−^ larvae reveals neuronal loss. ***a***, Histological assessment of 5 dpf *kif2a*^−/−^ and *kif2a^+/+^* larval brains. Six brain regions (from forebrain to hindbrain) were selected per genotype, as indicated in the diagram. ***b***, Comparison of the number of brain sections from forebrain to hindbrain (***a***, ***c–h***) between *kif2a^+/+^* and *kif2a*^−/−^ larvae. Data are represented as the mean ± SD. Significant values are noted as ****p ≤ 0.0001. ***c–h***, Coronal sections stained with H&E imaged at 20× magnification. CC, Cerebellar crest; CeP, cerebellar plate; Ch, chorda dorsalis; DIL, diffuse nucleus of inferior lobe; DT, dorsal thalamus; E, epiphysis; EmT, eminentia thalami; Ha, habenula; Hc, caudal hypothalamus; Hi, intermediate hypothalamus; Hyp, hypothalamus; lfb, lateral forebrain bundle; mlf, medial longitudinal fascicle; MO, medulla oblongata; OB, olfactory bulb; OC, otic capsule; OE, olfactory epithelium; P, pallium; PG, preglomerular complex; Pi, pigment; Po, preoptic region; PrT, pretectum; PTd, dorsal part of posterior tuberculum; PTv, ventral part of posterior tuberculum; S, subpallium; Teg, midbrain tegmentum; TeO, tectum opticum; Va, valvula cerebelli; VT, ventral thalamus. Bar graphs compare hematoxylin-positive stained nuclei between *kif2a^+/+^* and *kif2a*^−/−^ larvae. Data are represented as the mean ± SD. Significant values are noted as ****p ≤ 0.0001 and **p ≤ 0.01. Download Figure 5-1, TIF file.

Moreover, enlarged ventricles and cerebellar anomalies have been described as common features in *Kif2a*-null mice (Homma et al., 2003). To investigate whether microcephaly was caused by dysmorphologies in the brain and whether enlarged ventricles and cerebellar abnormalities were among the characteristics of our model, histologic examination of 5 dpf zebrafish brain tissue sections was performed (Fig. 5*e*, Extended Data Fig. 5-1*a–h*). Although zebrafish *kif2a*^−/−^ larvae did not present enlarged ventricles relative to the brain tissue, we found significantly fewer brain sections for *kif2a*^−/−^ larvae (75.5 ± 1.915, *n* = 4; *p* < 0.0001, Student’s *t* test) from forebrain to hindbrain (Fig. 5*e*,*f*, diagram) compared with their *kif2a^+/+^* siblings (85.0 ± 0.0, *n* = 4).

In addition, we observed that there were significantly fewer hematoxylin-positive stained nuclei detected in specific brain regions (Fig. 5*e*, *g* and *h*) of *kif2a*^−/−^ larvae (Fig. 5*g*, 955.3 ± 125.7, *n* = 4; *p *=* *0.0072, Student’s *t* test; Fig. 5*h*, 1556 ± 101.6, *n* = 4; *p *<* *0.0001, Student’s *t* test) than those in their *kif2a^+/+^* siblings (Fig. 5*g*, 1288 ± 109.8, *n* = 4; Fig. 5*h*, 2190 ± 46.99, *n* = 4). These findings are in line with a significantly smaller head size for *kif2a*^−/−^ larvae (Fig. 5*c*,*d*). Further examination showed that there were no cerebellar anomalies in the *kif2a* knock-out zebrafish brain. More particularly, all parts of the cerebellum such as valvula cerebelli and corpus cerebelli (cerebellar plate), together with the granular eminence (a caudolateral third part) were clearly present both in *kif2a^+/+^* and *kif2a*^−/−^ animals (Extended Data Fig. 5-1*g*,*h*; Mueller and Wullimann, 2016).

### Loss of *kif2a* is associated with neuronal cell proliferation defects and increased apoptosis

**Figure 6.**
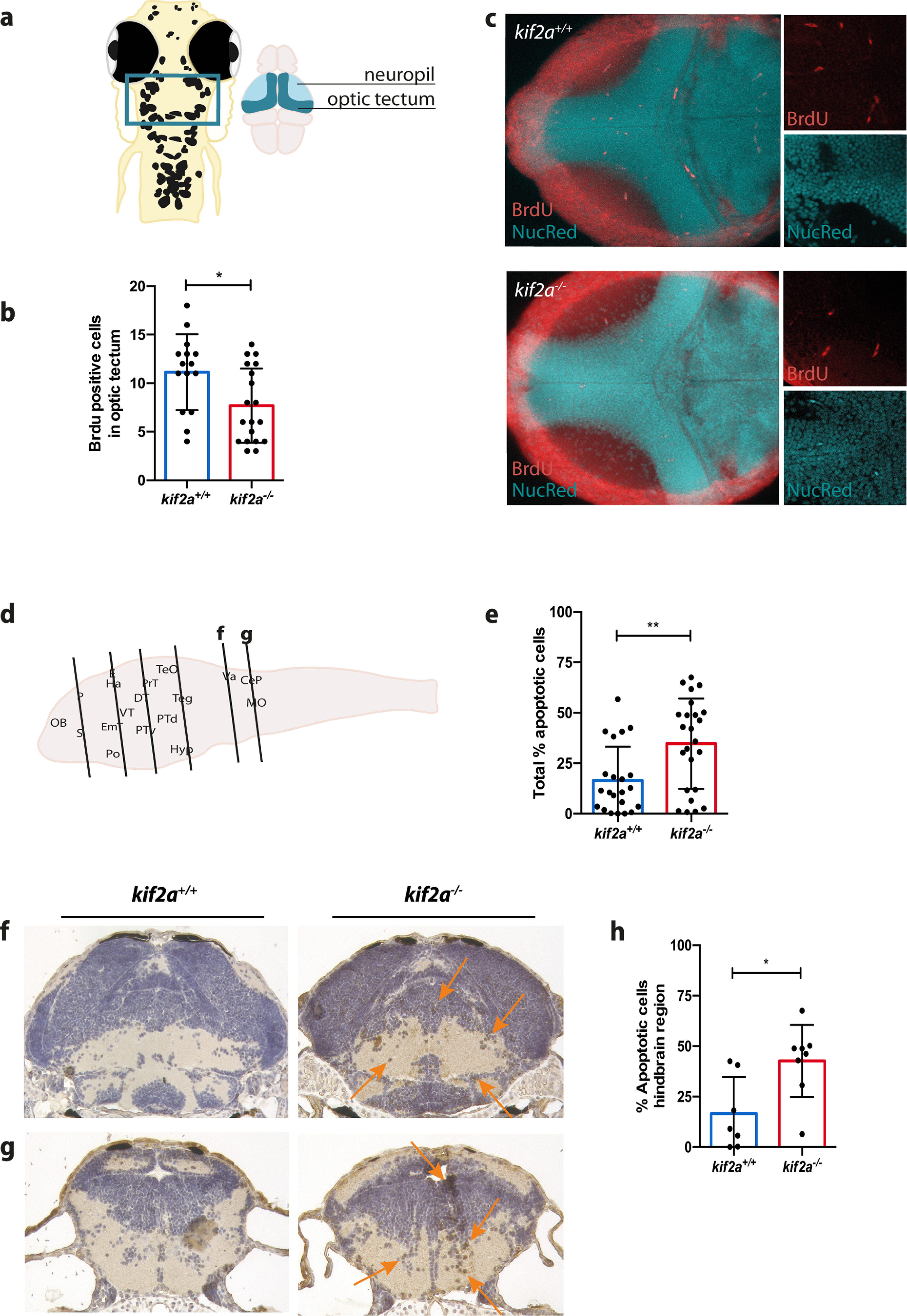
Neuronal cell proliferation defects and increased apoptosis in *kif2a*^−/−^ larvae. ***a***, Cell proliferation was measured in the optic tectum of 5 dpf *kif2a^+/+^* and *kif2a*^−/−^ larvae as indicated in the diagram. ***b***, Comparison of the number of proliferating cells in the optic tectum of *kif2a^+/+^* and *kif2a*^−/−^ larvae showing BrdU-positive cells in dorsal *z*-stacks. Data are represented as the mean ± SD. Significant values are noted as *p ≤ 0.1. ***c***, Representative *z*-stacks of optic tecta with BrdU and nuclei staining of *kif2a^+/+^* and *kif2a*^−/−^ larvae at 5 dpf. ***d***, Histologic assessment of 5 dpf *kif2a*^−/−^ and *kif2a^+/+^* larval brains. Six brain regions (from forebrain to hindbrain) were selected per genotype as indicated in the diagram. ***e***, Total amount of apoptotic cells in the brain of *kif2a^+/+^* and *kif2a*^−/−^ larvae. Data are represented as the mean ± SD. Significant values are noted as **p ≤ 0.01. ***f***, ***g***, Coronal sections stained against active caspase-3, imaged at 20× magnification. ***h***, Percentage of apoptotic cells in the hindbrain region of *kif2a^+/+^* and *kif2a*^−/−^ larvae. Data are represented as the mean ± SD. Significant values are noted as *p ≤ 0.05.

Having found that a loss of *kif2a* in zebrafish was associated with decreased head size and neuronal loss, we investigated whether this phenotype could be caused by defects in neuronal cell proliferation or excessive cell death in the brain. Whole-mount BrdU staining in 5 dpf zebrafish larval optic tectum (Fig. 6*a*) demonstrated that there was a significant decrease of proliferating cells in *kif2a*^−/−^ brains (7.684 ± 3.830, *n* = 19; *p *=* *0.0145, Student’s *t* test; Fig. 6*b*,*c*) when compared with their *kif2a^+/+^* siblings (11.13 ± 3.907, *n* = 15). Caspase-3 immunohistochemistry of 5 dpf larval sections, detecting early-stage apoptosis, showed an increase in the apoptotic cells for *kif2a*^−/−^ larvae (34.70 ± 22.31, *n* = 24; *p *=* *0.0033, Student’s *t* test; Fig. 6*e*) from forebrain to hindbrain (Fig. 6*d*, diagram) compared with their *kif2a^+/+^* siblings (16.49 ± 16.69, *n* = 22). In particular, as *kif2a* mRNA expression became restricted to hindbrain at 5 dpf (Fig. 2*c*), we checked whether elevated levels of apoptotic cells could be observed in sections obtained from this region (Fig. 6*d*, f and g, *f*, *g*). Indeed, we found a significant difference between regions of the hindbrain of *kif2a^+/+^* (16.61 ± 18.11, *n* = 7) and *kif2a*^−/−^ larvae (42.71 ± 17.80, *n* = 8; *p *=* *0.0147, Student’s *t* test; Fig. 6*h*).

## Discussion

Although mutations in tubulin and kinesin genes have been described in various syndromes associated with brain malformations and DRE (Bahi-Buisson and Cavallin, 2016; Poirier et al., 2013; Guerrini and Dobyns, 2014; Tian et al., 2016; Cavallin et al., 2017), and considerable preclinical/clinical knowledge has been obtained (Homma et al., 2003, 2018; Broix et al., 2018), the majority of patients with MCDs and associated DRE remain unresponsive to current pharmacological therapies (Papayannis et al., 2012; Represa, 2019). Therefore, a greater understanding of the mechanisms underlying MCDs is essential to developing more effective therapeutic agents against MCDs. Although mice are common choices for modeling human diseases, zebrafish offer a number of unique advantages of *in vitro* scalability in a vertebrate model, which is particularly valued in rapid CNS drug discovery (Stewart et al., 2015; Khan et al., 2017).

The aim of the present study was to advance our understanding of *KIF2A*-related MCDs and DRE by investigating the pathologic consequences of Kif2a loss of function in a novel zebrafish model. By targeting an early exon of *kif2a* sequence with CRISPR/Cas9, we generated an out-of-frame deletion leading to a premature stop codon, thus blocking downstream translation of Kif2a essential structural domains required for its activity, resulting in a total lack of Kif2a protein in our model. Correspondingly, missense variants described in patients with *KIF2A* mutations led to nonfunctional KIF2A protein (Poirier et al., 2013).

Our generated *kif2a* knock-out model possessed several similarities to *KIF2A* mouse models that recapitulated many features of the human phenotype. In particular, *kif2a*^−/−^ zebrafish larvae had reduced head size compared with their *kif2a^+/+^* siblings. This neuroanatomical anomaly was supported by neuronal loss in the brain because of a decrease in cell proliferation and increased levels of apoptotic cells in the brains of *kif2a*^−/−^ mutants. Correspondingly, Gilet et al. (2020) found that abnormal neuronal migration, large-scale apoptosis, and increased p53 levels during early stages in the entire mouse cortex were likely responsible for the observed microcephaly in *KIF2A^+/H321D^* mice. Programmed cell death was shown to be increased in many mouse models of microcephaly (Poulton et al., 2011; Little and Dwyer, 2019), and, in contrast, a reduction of apoptosis leads to increased brain size (Kuida et al., 1998). Additionally, previous studies have already reported the correlation between KIF2A inactivation and induced apoptosis in cancer cells (Zhang et al., 2016, 2019; Li et al., 2019). The silencing of KIF2A by siRNA resulted in a decrease of PI3 kinase/Akt expression and downstream proteins, a key signaling pathway involved in the regulation of cell proliferation and growth, and ultimately in the initiation of apoptosis (Wang et al., 2014; Zhang et al., 2020). In addition, the neuroprotective role of the PI3 kinase/Akt signaling pathway in zebrafish has already been elucidated, emphasizing its function in the attainment of normal brain size during zebrafish embryogenesis (Chen et al., 2017).

Moreover, *Kif2a*-cKO mice (Homma et al., 2018) and *KIF2A^+/H321D^* mice (Gilet et al., 2020) presented with behavioral deficiencies and susceptibility to epilepsy. Similarly, *kif2a*^−/−^ larvae showed habituation learning deficits as they were not able to respond to the trained stimulus during the learning period, suggesting inefficient storage of information and memory capacity. These data are in line with memory decay and learning problems, hallmarks of developmental delay and intellectual disability observed in human subjects presenting with *KIF2A* mutations (Poirier et al., 2013; Tian et al., 2016; Cavallin et al., 2017). We believe that defects in neuronal cell proliferation and increased apoptosis in *kif2a*^−/−^ mutants may have direct effects on key structures in the brain playing a crucial role in memory. Furthermore, DRE was one of the disease symptoms among patients with mutations in *KIF2A* (Poirier et al., 2013; Tian et al., 2016; Cavallin et al., 2017). In contrast to the spontaneous epileptiform events observed in *Kif2a*-cKO mice during postnatal week 5 (Homma et al., 2018), *kif2a* mutant larvae only exhibited epileptiform events when incubated with a subthreshold dose of a convulsant, PTZ. These findings were supported by the study from Gilet et al. (2020), where *KIF2A^+/H321D^* mice also presented seizure susceptibility after PTZ administration.

Albeit our *kif2a* zebrafish model recapitulated many phenotypic hallmarks identified in mouse models and human patients with *KIF2A* mutations, neuroanatomical brain abnormalities described in Kif2a mice models (Homma et al., 2003; Gilet et al., 2020) could not be addressed in our model. This could possibly be because of the fact that the brain ventricular system in mammals is different from that of teleosts (Korzh, 2018), and, although that dorsal pallium likely contains structures homologous to the mammalian hippocampus (Cheng et al., 2014; Saleem and Kannan, 2018), a precise functional mapping of these brain territories is still largely missing. Also, in contrast to the hyperactivity documented in *KIF2A* mouse models (Homma et al., 2018; Gilet et al., 2020), we observed a reduced locomotor activity in *kif2a*^−/−^ larvae. Although epilepsy is often linked to increased locomotor activity and thus hyperexcitability, various genetic epilepsy zebrafish models have already reported hypoactivity (Scheldeman et al., 2017; Swaminathan et al., 2018; Prentzell et al., 2021). More particularly, some genetic epilepsy mouse models showed hypoactivity as well (Gu et al., 2019), whereas patients are often hyperactive (Pelc et al., 2008). Although light–dark transitions mainly result in hyperactivity (Basnet et al., 2019) because of a boost in the stress or anxiety levels in zebrafish larvae, locomotor behavior strongly depends on precise CNS development and proper functioning of the brain pathways. Our results show that a failure of any of these factors may be responsible for the observed decrease in locomotor activity (Bilotta, 2000; MacPhail et al., 2009; Vignet et al., 2013; Basnet et al., 2019).

Given the fact that *Kif2a* KO mice died within a day of birth (Homma et al., 2003) and *Kif2a*-cKO mice died by the end of week 6 (Homma et al., 2018), and because *kif2a* was selectively expressed in the developing brain during embryogenesis, we hypothesized that our (maternal zygotic) *kif2a* KO zebrafish larvae would die prematurely. Interestingly, *kif2a*^−/−^ zebrafish larvae survived until adulthood and were fertile, and thus its offspring, hence excluding that survival was essentially regulated by compensatory effects of maternal *kif2a* transcripts or Kif2a protein. Possibly, some genetic compensation mechanisms could be involved; however, we did not observe any upregulation originating from a direct *kinesin-13* family member. We therefore speculate that additional adaptive compensation by other genes may apply; however, such investigations would require the use of transcriptomics, which could provide an elaborate gene ontology enrichment map of upregulated or downregulated genes. Nonetheless, it is also possible that Kif2a function in zebrafish is less crucial than in mice and not necessarily compensated per se. In addition, we observed that *kif2a*^−/−^ zebrafish obtained from heterozygous parents had no behavioral phenotype compared with *kif2a*^−/−^ zebrafish derived from homozygous fish. Given the substantial role of maternal mRNA in zebrafish oocytes, maternal *kif2a* might continue to perform its function in early larval development (even after zygotic gene expression initiation), leaving *kif2a*^−/−^ larvae from heterozygous parents unaffected (Pelegri, 2003).

Together, we demonstrated that our *kif2a*^−/−^ zebrafish model mimicked well the important aspects of the phenotype observed in mice models being reminiscent of the phenotype in patients with *KIF2A* mutations. Our results highlight the value of our novel *kif2a* KO model in understanding of the pathologic consequences of KIF2A loss of function. Importantly, since our model does not require tedious genotyping procedures, it could possibly be deployed in medium- to high-throughput MCD/DRE-relevant drug screens and hence used as a high-throughput screening model in a future search for novel and more effective therapies.

## Synthesis

Reviewing Editor: Katalin Toth, University of Ottawa Decisions are customarily a result of the Reviewing Editor and the peer reviewers coming together and discussing their recommendations until a consensus is reached. When revisions are invited, a fact-based synthesis statement explaining their decision and outlining what is needed to prepare a revision will be listed below. The following reviewer(s) agreed to reveal their identity: Eric Samarut, Nobutaka Hirokawa.

The authors have significantly improved the manuscript. However, there is still one outstanding issue. The authors write in their rebuttal letter that “ Concerning the possible changes in arborization and wiring of zebrafish kif2a-/- larvae, we

believe these experiments are not really essential for understanding our kif2a story.” If this is their belief this should be clearly argued and explained in their rebuttal. Reviewer #2 still would like to see a more complete answer to his/her question. As it is clear from Rev.#2’s comments the response to this question was not adequate. The authors have two choices, they can either argue why this issue is not critical for their paper, or they can provide the data requested by Rev. #2.

Rev. #2:

In this revision the authors have improved their manuscript along with the reviewers’ previous suggestions but the brain wiring issue. The reason why this reviewer has previously recommended to assess the brain wiring/neurite branching pattern was that it has been significantly disorganized in mouse KIF2A knockout and conditional knockout models from Hirokawa and colleagues had done before. They had shown that KIF2A deficient neurons have exhibited excessive axon collateral extension due to impaired microtubule depolymerization, which caused recurrent synapses as the structural basis underlying this epilepsy-like phenotype. Thus, because this zebrafish model also tended to cause epilepsy, it is natural to see how their axonal arborization looked like for phenotype characterization. Accordingly, this reviewer strongly recommends the authors to provide these cytological analyses in this paper. Also, in Fig. 1b, it is weird to artificially split the sequence electropherogram into two regions only for the presentation purpose. Please show the original data without splitting the mutant one.

## References

[B1] Afrikanova T, Serruys AS, Buenafe OE, Clinckers R, Smolders I, de Witte PA, Crawford AD, Esguerra CV (2013) Validation of the zebrafish pentylenetetrazol seizure model: locomotor versus electrographic responses to antiepileptic drugs. PLoS One 8:e54166. 10.1371/journal.pone.0054166 23342097 PMC3544809

[B2] Ali I, Yang WC (2020) The functions of kinesin and kinesin-related proteins in eukaryotes. Cell Adh Migr 14:139–152. 10.1080/19336918.2020.1810939 32842864 PMC7513868

[B3] Bahi-Buisson N, Cavallin M (2016) Tubulinopathies overview. In: GeneReviews (, Adam MP, Ardinger HH, Pagon RA, Wallace SE, Bean LJH, Mirzaa G, Amemiya A, eds). Seattle, WA: University of Washington; 1993-2017. 27010057

[B4] Basnet RM, Zizioli D, Taweedet S, Finazzi D, Memo M (2019) Zebrafish larvae as a behavioral model in neuropharmacology. Biomedicines 7:23. 10.3390/biomedicines7010023 PMC6465999 30917585

[B5] Bilotta J (2000) Effects of abnormal lighting on the development of zebrafish visual behavior. Behav Brain Res 116:81–87. 10.1016/s0166-4328(00)00264-3 11090887

[B6] Broix L, Asselin L, Silva CG, Ivanova EL, Tilly P, Gilet JG, Lebrun N, Jagline H, Muraca G, Saillour Y, Drouot N, Reilly ML, Francis F, Benmerah A, Bahi-Buisson N, Belvindrah R, Nguyen L, Godin JD, Chelly J, Hinckelmann M-V (2018) Ciliogenesis and cell cycle alterations contribute to KIF2A-related malformations of cortical development. Hum Mol Genet 27:224–238. 10.1093/hmg/ddx384 29077851

[B7] Buchsbaum IY, Cappello S (2019) Neuronal migration in the CNS during development and disease: insights from in vivo and in vitro models. Development 146:dev163766. 10.1242/dev.163766 30626593

[B8] Cavallin M, Bijlsma EK, El Morjani A, Moutton S, Peeters EA, Maillard C, Pedespan JM, Guerrot AM, Drouin-Garaud V, Coubes C, Genevieve D, Bole-Feysot C, Fourrage C, Steffann J, Bahi-Buisson N (2017) Recurrent KIF2A mutations are responsible for classic lissencephaly. Neurogenetics 18:73–79. 10.1007/s10048-016-0499-8 27747449

[B9] Chen S, Liu Y, Rong X, Li Y, Zhou J, Lu L (2017) Neuroprotective role of the PI3 kinase/Akt signaling pathway in zebrafish. Front Endocrinol (Lausanne) 8:21. 10.3389/fendo.2017.00021 28228749 PMC5296330

[B10] Cheng RK, Jesuthasan SJ, Penney TB (2014) Zebrafish forebrain and temporal conditioning. Philos Trans R Soc Lond B Biol Sci 369:20120462. 10.1098/rstb.2012.0462 24446496 PMC3895987

[B11] Desikan RS, Barkovich AJ (2016) Malformations of cortical development. Ann Neurol 80:797–810. 10.1002/ana.24793 27862206 PMC5177533

[B12] Diaz AL, Gleeson JG (2009) The molecular and genetic mechanisms of neocortex development. Clin Perinatol 36:503–512. 10.1016/j.clp.2009.06.008 19732610 PMC2771632

[B13] Gilet JG, Ivanova EL, Trofimova D, Rudolf G, Meziane H, Broix L, Drouot N, Courraud J, Skory V, Voulleminot P, Osipenko M, Bahi-Buisson N, Yalcin B, Birling MC, Hinckelmann MV, Kwok BH, Allingham JS, Chelly J (2020) Conditional switching of KIF2A mutation provides new insights into cortical malformation pathogeny. Hum Mol Genet 29:766–784. 10.1093/hmg/ddz316 31919497 PMC7104682

[B14] Gu B, Zhu M, Glass MR, Rougié M, Nikolova VD, Moy SS, Carney PR, Philpot BD (2019) Cannabidiol attenuates seizures and EEG abnormalities in Angelman syndrome model mice. J Clin Invest 129:5462–5467. 10.1172/JCI130419 31503547 PMC6877312

[B15] Guerrini R, Dobyns WB (2014) Malformations of cortical development: clinical features and genetic causes. Lancet Neurol 13:710–726. 10.1016/S1474-4422(14)70040-7 24932993 PMC5548104

[B16] Homma N, Takei Y, Tanaka Y, Nakata T, Terada S, Kikkawa M, Noda Y, Hirokawa N (2003) Kinesin superfamily protein 2A (KIF2A) functions in suppression of collateral branch extension. Cell 114:229–239. 10.1016/s0092-8674(03)00522-1 12887924

[B17] Homma N, Zhou R, Naseer MI, Chaudhary AG, Al-Qahtani MH, Hirokawa N (2018) KIF2A regulates the development of dentate granule cells and postnatal hippocampal wiring. Elife 7:e30935. 10.7554/eLife.30935 29313800 PMC5811213

[B18] Hruscha A, Krawitz P, Rechenberg A, Heinrich V, Hecht J, Haass C, Schmid B (2013) Efficient CRISPR/Cas9 genome editing with low off-target effects in zebrafish. Development 140:4982–4987. 10.1242/dev.099085 24257628

[B19] Hwang WY, Fu Y, Reyon D, Maeder ML, Tsai SQ, Sander JD, Peterson RT, Yeh JR, Joung JK (2013) Efficient genome editing in zebrafish using a CRISPR-Cas system. Nat Biotechnol 31:227–229. 10.1038/nbt.2501 23360964 PMC3686313

[B20] Kalueff AV, Stewart AM, Gerlai R (2014) Zebrafish as an emerging model for studying complex brain disorders. Trends Pharmacol Sci 35:63–75. 10.1016/j.tips.2013.12.002 24412421 PMC3913794

[B21] Khan KM, Collier AD, Meshalkina DA, Kysil EV, Khatsko SL, Kolesnikova T, Morzherin YY, Warnick JE, Kalueff AV, Echevarria DJ (2017) Zebrafish models in neuropsychopharmacology and CNS drug discovery. Br J Pharmacol 174:1925–1944. 10.1111/bph.13754 28217866 PMC5466539

[B22] Korzh V (2018) Development of brain ventricular system. Cell Mol Life Sci 75:375–383. 10.1007/s00018-017-2605-y 28780589 PMC5765195

[B23] Kuida K, Haydar TF, Kuan CY, Gu Y, Taya C, Karasuyama H, Su MS, Rakic P, Flavell RA (1998) Reduced apoptosis and cytochrome c-mediated caspase activation in mice lacking caspase 9. Cell 94:325–337. 10.1016/s0092-8674(00)81476-2 9708735

[B24] Leventer RJ, Phelan EM, Coleman LT, Kean MJ, Jackson GD, Harvey AS (1999) Clinical and imaging features of cortical malformations in childhood. Neurology 53:715–715. 10.1212/wnl.53.4.715 10489031

[B25] Leventer RJ, Guerrini R, Dobyns WB (2008) Malformations of cortical development and epilepsy. Dialogues Clin Neurosci 10:47–62. 18472484 10.31887/DCNS.2008.10.1/rjleventer PMC3181860

[B26] Li X, Shu K, Wang Z, Ding D (2019) Prognostic significance of KIF2A and KIF20A expression in human cancer: a systematic review and meta-analysis. Medicine (Baltimore) 98:e18040. 10.1097/MD.0000000000018040 31725680 PMC6867763

[B27] Little JN, Dwyer ND (2019) p53 deletion rescues lethal microcephaly in a mouse model with neural stem cell abscission defects. Hum Mol Genet 28:434–447. 10.1093/hmg/ddy350 30304535 PMC6337704

[B28] MacPhail RC, Brooks J, Hunter DL, Padnos B, Irons TD, Padilla S (2009) Locomotion in larval zebrafish: influence of time of day, lighting and ethanol. Neurotoxicology 30:52–58. 10.1016/j.neuro.2008.09.011 18952124

[B29] Maor-Nof M, Homma N, Raanan C, Nof A, Hirokawa N, Yaron A (2013) Axonal pruning is actively regulated by the microtubule-destabilizing protein kinesin superfamily protein 2A. Cell Rep 3:971–977. 10.1016/j.celrep.2013.03.005 23562155

[B30] Miki H, Okada Y, Hirokawa N (2005) Analysis of the kinesin superfamily: insights into structure and function. Trends Cell Biol 15:467–476. 10.1016/j.tcb.2005.07.006 16084724

[B31] Mueller T, Wullimann M (2016) Atlas of early zebrafish brain development, Ed 2. London: Elsevier.

[B32] Nelson JC, Witze E, Ma Z, Ciocco F, Frerotte A, Randlett O, Foskett JK, Granato M (2020) Acute regulation of habituation learning via posttranslational palmitoylation. Curr Biol 30:2729–2738.e4. 10.1016/j.cub.2020.05.016 32502414 PMC8446937

[B33] Pang T, Atefy R, Sheen V (2008) Malformations of cortical development. Neurologist 14:181–191. 10.1097/NRL.0b013e31816606b9 18469675 PMC3547618

[B34] Papayannis CE, Consalvo D, Kauffman MA, Seifer G, Oddo S, D'Alessio L, Saidon P, Kochen S (2012) Malformations of cortical development and epilepsy in adult patients. Seizure 21:377–384. 10.1016/j.seizure.2012.03.009 22513002

[B35] Pelc K, Cheron G, Dan B (2008) Behavior and neuropsychiatric manifestations in Angelman syndrome. Neuropsychiatr Dis Treat 4:577–584. 10.2147/ndt.s2749 18830393 PMC2526368

[B36] Pelegri F (2003) Maternal factors in zebrafish development. Dev Dyn 228:535–554. 10.1002/dvdy.10390 14579391

[B37] Poirier K, Lebrun N, Broix L, Tian G, Saillour Y, Boscheron C, Parrini E, Valence S, Pierre BS, Oger M, Lacombe D, Geneviève D, Fontana E, Darra F, Cances C, Barth M, Bonneau D, Bernadina BD, N’guyen S, Gitiaux C, et al. (2013) Mutations in TUBG1, DYNC1H1, KIF5C and KIF2A cause malformations of cortical development and microcephaly. Nat Genet 45:639–647. 10.1038/ng.2613 23603762 PMC3826256

[B38] Poulton CJ, Schot R, Kia SK, Jones M, Verheijen FW, Venselaar H, de Wit MC, de Graaff E, Bertoli-Avella AM, Mancini GM (2011) Microcephaly with simplified gyration, epilepsy, and infantile diabetes linked to inappropriate apoptosis of neural progenitors. Am J Hum Genet 89:265–276. 10.1016/j.ajhg.2011.07.006 21835305 PMC3155199

[B239] Prentzell MT, Rehbein U, Cadena Sandoval M, De Meulemeester A-S, Baumeister R, Brohée L, Berdel B, Bockwoldt M, Carroll B, Chowdhury SR, von Deimling A, Demetriades C, Figlia G, de Araujo MEG, Heberle AM, Heiland I, Holzwarth B, Huber LA, Jaworski J, Kedra M, et al. (2021) G3BPs tether the TSC complex to lysosomes and suppress mTORC1 signaling. Cell 184:655–674.e27. 10.1016/j.cell.2020.12.024 33497611 PMC7868890

[B40] Represa A (2019) Why malformations of cortical development cause epilepsy. Front Neurosci 13:250–250. 10.3389/fnins.2019.00250 30983952 PMC6450262

[B41] Saleem S, Kannan RR (2018) Zebrafish: an emerging real-time model system to study Alzheimer's disease and neurospecific drug discovery. Cell Death Discov 4:45. 10.1038/s41420-018-0109-7 PMC6170431 30302279

[B42] Scheldeman C, Mills JD, Siekierska A, Serra I, Copmans D, Iyer AM, Whalley BJ, Maes J, Jansen AC, Lagae L, Aronica E, de Witte PAM (2017) mTOR-related neuropathology in mutant tsc2 zebrafish: phenotypic, transcriptomic and pharmacological analysis. Neurobiol Dis 108:225–237. 10.1016/j.nbd.2017.09.004 28888969

[B39] Siekierska A, Stamberger H, Deconinck T, Oprescu SN, Partoens M, Zhang Y, Sourbron J, Adriaenssens E, Mullen P, Wiencek P, Hardies K, Lee J-S, Giong H-K, Distelmaier F, Elpeleg O, Helbig KL, Hersh J, Isikay S, Jordan E, Karaca E, et al. (2019) Biallelic VARS variants cause developmental encephalopathy with microcephaly that is recapitulated in var knockout zebrafish. Nat Commun 10:708. 30755616 10.1038/s41467-018-07953-w PMC6372652

[B43] Stewart AM, Gerlai R, Kalueff AV (2015) Developing highER-throughput zebrafish screens for in-vivo CNS drug discovery. Front Behav Neurosci 9:14. 10.3389/fnbeh.2015.00014 25729356 PMC4325915

[B44] Sun D, Zhou X, Yu HL, He XX, Guo WX, Xiong WC, Zhu XJ (2017) Regulation of neural stem cell proliferation and differentiation by Kinesin family member 2a. PLoS One 12:e0179047. 10.1371/journal.pone.0179047 28591194 PMC5462413

[B45] Swaminathan A, Hassan-Abdi R, Renault S, Siekierska A, Riché R, Liao M, de Witte PAM, Yanicostas C, Soussi-Yanicostas N, Drapeau P, Samarut É (2018) Non-canonical mTOR-independent role of DEPDC5 in regulating GABAergic network development. Curr Biol 28:1924–1937.e5. 10.1016/j.cub.2018.04.061 29861134

[B46] Tian G, Cristancho AG, Dubbs HA, Liu GT, Cowan NJ, Goldberg EM (2016) A patient with lissencephaly, developmental delay, and infantile spasms, due to de novo heterozygous mutation of KIF2A. Mol Genet Genomic Med 4:599–603. 10.1002/mgg3.236 27896282 PMC5118204

[B47] Tiraboschi E, Martina S, van der Ent W, Grzyb K, Gawel K, Cordero‐Maldonado ML, Poovathingal SK, Heintz S, Satheesh SV, Brattespe J, Xu J, Suster M, Skupin A, Esguerra CV (2020) New insights into the early mechanisms of epileptogenesis in a zebrafish model of Dravet syndrome. Epilepsia 61:549–560. 10.1111/epi.16456 32096222

[B48] Vignet C, Bégout ML, Péan S, Lyphout L, Leguay D, Cousin X (2013) Systematic screening of behavioral responses in two zebrafish strains. Zebrafish 10:365–375. 10.1089/zeb.2013.0871 23738739

[B49] Wang K, Lin C, Wang C, Shao Q, Gao W, Song B, Wang L, Song X, Qu X, Wei F (2014) Silencing Kif2a induces apoptosis in squamous cell carcinoma of the oral tongue through inhibition of the PI3K/Akt signaling pathway. Mol Med Rep 9:273–278. 10.3892/mmr.2013.1804 24248467

[B50] Wolman MA, Jain RA, Liss L, Granato M (2011) Chemical modulation of memory formation in larval zebrafish. Proc Natl Acad Sci U S A 108:15468–15473. 10.1073/pnas.1107156108 21876167 PMC3174630

[B51] Zhang Q, Lu D, Liu W, Ye S, Guo H, Liao T, Chen C (2019) Effects of KIF2A on the prognosis of nasopharyngeal carcinoma and nasopharyngeal carcinoma cells. Oncol Lett 18:2718–2723. 10.3892/ol.2019.10597 31452750 PMC6676658

[B52] Zhang X, Ma C, Wang Q, Liu J, Tian M, Yuan Y, Li X, Qu X (2016) Role of KIF2A in the progression and metastasis of human glioma. Mol Med Rep 13:1781–1787. 10.3892/mmr.2015.4700 26707290

[B53] Zhang X, Wang Y, Liu X, Zhao A, Yang Z, Kong F, Sun L, Yu Y, Jiang L (2020) KIF2A promotes the progression via AKT signaling pathway and is upregulated by transcription factor ETV4 in human gastric cancer. Biomed Pharmacother 125:109840. 10.1016/j.biopha.2020.109840 32106376

