# Supplementary figures and images for: Modeling Neurodevelopmental Disorders and Epilepsy Caused by Loss of Function of kif2a in Zebrafish
Source: eNeuro. 2021 Sep 7;8(5):ENEURO.0055-21.2021. doi: 10.1523/ENEURO.0055-21.2021 (PMC8425962; doi:10.1523/ENEURO.0055-21.2021)

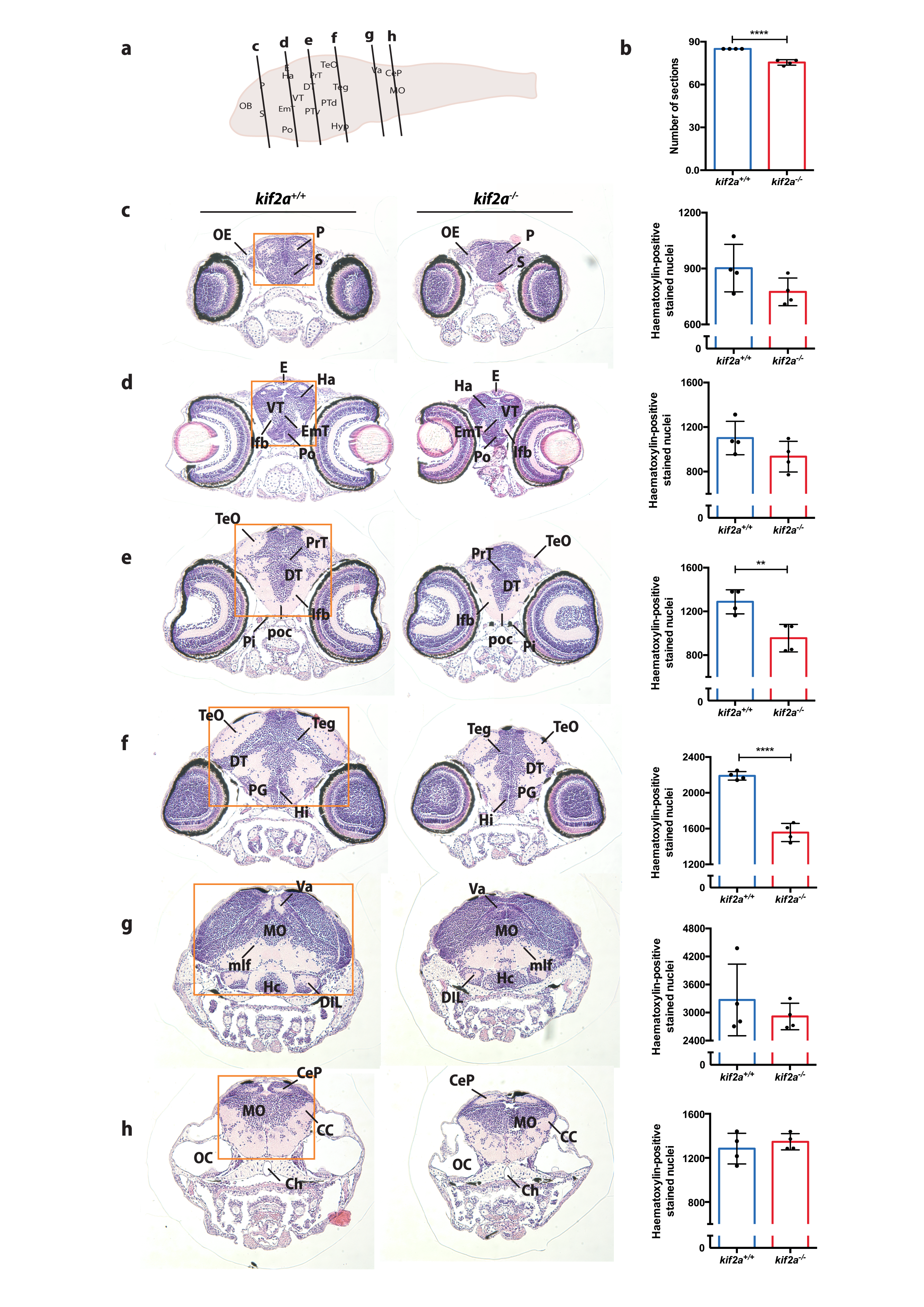

Supplement: Figure 5-1 — H&E histological staining of kif2a−/− larvae reveals neuronal loss. a, Histological assessment of 5 dpf kif2a−/− and kif2a+/+ larval brains. Six brain regions (from forebrain to hindbrain) were selected per genotype, as indicated in the diagram. b, Comparison of the number of brain sections from forebrain to hindbrain (a, c–h) between kif2a+/+ and kif2a−/− larvae. Data are represented as the mean ± SD. Significant values are noted as ****p ≤ 0.0001. c–h, Coronal sections stained with H&E imaged at 20× magnification. CC, Cerebellar crest; CeP, cerebellar plate; Ch, chorda dorsalis; DIL, diffuse nucleus of inferior lobe; DT, dorsal thalamus; E, epiphysis; EmT, eminentia thalami; Ha, habenula; Hc, caudal hypothalamus; Hi, intermediate hypothalamus; Hyp, hypothalamus; lfb, lateral forebrain bundle; mlf, medial longitudinal fascicle; MO, medulla oblongata; OB, olfactory bulb; OC, otic capsule; OE, olfactory epithelium; P, pallium; PG, preglomerular complex; Pi, pigment; Po, preoptic region; PrT, pretectum; PTd, dorsal part of posterior tuberculum; PTv, ventral part of posterior tuberculum; S, subpallium; Teg, midbrain tegmentum; TeO, tectum opticum; Va, valvula cerebelli; VT, ventral thalamus. Bar graphs compare hematoxylin-positive stained nuclei between kif2a+/+ and kif2a−/− larvae. Data are represented as the mean ± SD. Significant values are noted as ****p ≤ 0.0001 and **p ≤ 0.01. Download Figure 5-1, TIF file. [file enu-eN-NWR-0055-21-s05.tif]
